# Comprehensive analysis of the *Ppatg3* mutant reveals that autophagy plays important roles in gametophore senescence in *Physcomitrella patens*

**DOI:** 10.1186/s12870-020-02651-6

**Published:** 2020-09-23

**Authors:** Zexi Chen, Wenbo Wang, Xiaojun Pu, Xiumei Dong, Bei Gao, Ping Li, Yanxia Jia, Aizhong Liu, Li Liu

**Affiliations:** 1 Department of Economic Plants and Biotechnology, Yunnan Key Laboratory for Wild Plant Resources, Kunming Institute of Botany, Chinese Academy of Sciences, Kunming, 650204 China; 2 University of the Chinese Academy of Sciences, Beijing, 100049 China; 3 Sino-Danish College, University of Chinese Academy of Sciences, Beijing, 100049 China; 4 State Key Laboratory of Agrobiotechnology, The Chinese University of Hong Kong, Hong Kong, 999077 China; 5 Germplasm Bank of Wild Species, Kunming Institute of Botany, Chinese Academy of Sciences, Kunming, 650204 China; 6 Key Laboratory for Forest Resources Conservation and Utilization in the Southwest Mountains of China Ministry of Education, Southwest Forestry University, Kunming, 650204 China; 7 State Key Laboratory of Biocatalysis and Enzyme Engineering, Hubei Collaborative Innovation Center for Green Transformation of Bio-Resources, Hubei Key Laboratory of Industrial Biotechnology, School of Life Sciences, Hubei University, Wuhan, 430062 China

**Keywords:** Autophagy defect, ATG, C/N ratio, Fatty acid, Chloroplast plastoglobuli, Premature senescence, Moss

## Abstract

**Background:**

Autophagy is an evolutionarily conserved system for the degradation of intracellular components in eukaryotic organisms. Autophagy plays essential roles in preventing premature senescence and extending the longevity of vascular plants. However, the mechanisms and physiological roles of autophagy in preventing senescence in basal land plants are still obscure.

**Results:**

Here, we investigated the functional roles of the autophagy-related gene *PpATG3* from *Physcomitrella patens* and demonstrated that its deletion prevents autophagy. In addition, *Ppatg3* mutant showed premature gametophore senescence and reduced protonema formation compared to wild-type (WT) plants under normal growth conditions. The abundance of nitrogen (N) but not carbon (C) differed significantly between *Ppatg3* mutant and WT plants, as did relative fatty acid levels. In vivo protein localization indicated that PpATG3 localizes to the cytoplasm, and in vitro Y2H assays confirmed that PpATG3 interacts with PpATG7 and PpATG12. Plastoglobuli (PGs) accumulated in *Ppatg3*, indicating that the process that degrades damaged chloroplasts in senescent gametophore cells was impaired in this mutant. RNA-Seq uncovered a detailed, comprehensive set of regulatory pathways that were affected by the autophagy mutation.

**Conclusions:**

The autophagy-related gene *PpATG3* is essential for autophagosome formation in *P. patens*. Our findings provide evidence that autophagy functions in N utilization, fatty acid metabolism and damaged chloroplast degradation under non-stress conditions. We identified differentially expressed genes in *Ppatg3* involved in numerous biosynthetic and metabolic pathways, such as chlorophyll biosynthesis, lipid metabolism, reactive oxygen species removal and the recycling of unnecessary proteins that might have led to the premature senescence of this mutant due to defective autophagy. Our study provides new insights into the role of autophagy in preventing senescence to increase longevity in basal land plants.

## Background

Autophagy is an evolutionarily conserved, ubiquitous process in eukaryotic cells that degrades damaged or toxic intracellular components for recycling to maintain essential cellular functions and life activities [1–3]. In plants, autophagy contributes to nutrient use efficiency and energy metabolism and is upregulated during senescence to promote cellular homeostasis and longevity [4–6]. Two types of autophagy pathways have been identified in plants: macroautophagy and microautophagy [7]. Macroautophagy, which had been extensively studied, is regulated by *AuTophaGy* (*ATG*) genes, whose expression results in the formation of a double-membrane organelle known as the autophagosome [2]. Bulk cytosolic components, including organelle fragments and macromolecules, are then transferred into the vacuole via fusion with the autophagosome and are subsequently degraded by lytic enzymes within the vacuole. We use the term ‘autophagy’ hereafter to refer specifically to macroautophagy. To date, at least 30 ATG proteins had been identified in yeast (*Saccharomyces cerevisiae*), which can be divided into several functional classes: a) the ATG1-ATG13 kinase complex; b) ATG9 and ATG9-associated proteins; c) the phosphatidylinositol 3-kinase complex; and d) two ubiquitin-like conjugation systems mediated by ATG8 or ATG12 [8]. Most of these proteins have homologs in plants. Autophagy plays multiple physiological roles in plants, functioning in processes such as biotic and abiotic stress responses [9, 10], anther development [11], leaf starch degradation [12], lipid/fatty acid homeostasis and turnover [11, 13–15], damaged chloroplast degradation [16, 17], soluble/aggregated protein degradation [18] and senescence [2, 19]. ATG3 is an E2-like enzyme involved in the ATG8 and phosphatidylethanolamine (PE) conjugation system during autophagosome formation [20]. Based on the crystal structure of *S. cerevisiae* ATG3, cysteine 234 (Cys-234) is the active residue that is important for the lipidization reaction of ATG8-PE conjugation [21]. Autophagosome formation is defective in *ATG3* mutant in yeast [22] and *Toxoplasma* [23], and autophagic activity was enhanced by overexpressing *ATG3* in tobacco [24].

Autophagy is a fundamental factor in cell longevity and senescence in eukaryotes, especially plants [2, 4]. The recycling and remobilization of nutrients, including carbon (C) and nitrogen (N), are crucial for plant survival and adaptation, especially under nutrient-limiting conditions [25]. Recent reports in *Arabidopsis thaliana* (Arabidopsis) revealed that autophagy is important for N-remobilization efficiency [26–28] and controls the C/N ratio [29]. However, to date, most studies in Arabidopsis on the roles of autophagy in nitrogen utilization and senescence were conducted under nutrient starvation or abiotic stress conditions, and few studies have focused on these processes under normal growth conditions. Moreover, recent studies suggested that autophagy plays important roles in lipid/fatty acid metabolism [11], composition [13] and turnover [14] in several vascular plants, although whether autophagy affects fatty acids in basal land plants is unknown. Even though autophagy is known to be essential for C/N status and lipid/fatty acid metabolism in plants, the details of the autophagy regulatory machinery are mostly unknown.

*Physcomitrella patens*, a basal land plant commonly used for developmental biology research, had been used to study autophagy during senescence in the dark [30] and during gamete differentiation [31]. However, to date, only two autophagy genes, *ATG5* and *ATG7*, have been identified and studied in *P. patens*. Further elucidation of the regulatory pathway of *ATG*s in moss would increase our understanding of the roles of autophagy in plant development. In the current study, we analyzed *ATG3* knockout *P. patens* lines under normal growth conditions. The gametophores of the mutant displayed early-senescence symptoms, including yellowing, impaired photosynthesis, reduced chlorophyll levels, the accumulation of chloroplast plastoglobuli (PGs) and differential expression of senescence**-**associated genes (SAGs) under normal growth conditions. Analysis of whole-plant C/N ratios and fatty acid contents revealed that autophagy plays essential roles in N-utilization efficiency and fatty acid metabolism in *P. patens* gametophores. In addition, we performed comprehensive RNA-Seq analysis to provide insight into the role of autophagy in gametophore senescence in *P. patens*. Our study provides evidence for the role of autophagy in N utilization, fatty acid/lipid metabolism, damaged chloroplast degradation, reactive oxygen species (ROS) removal and recycling of unnecessary proteins under non-stress conditions to prevent senescence and enhance cell longevity in the basal land plant *P. patens*.

## Results

### Identification of *ATG3* from *P. patens*

The 924-bp *PpATG3* coding sequence contains 9 exons and is almost the same size as Arabidopsis *ATG3* (AT5G61500, 942 bp, with 9 exons). Protein sequence alignment revealed both conservation and divergence of the three primary functional domains of ATG3 proteins in *P. patens* vs. *Klebsormidium nitens*, *Mesotaenium endlicherianum*, *Anthoceros angustus*, *Marchantia polymorpha*, *Brachypodium distachyon*, *A. thaliana*, *S. cerevisiae*, *Mus musculus* and *Homo sapiens* (Additional file 1A). Two of these domains (Autophagy_act_C and Autophagy_C) showed high levels of conservation, while the third (Autophagy_N) was weakly conserved. Notably, the Autophagy_C domain was missing in the subaerial green alga *Mesotaenium endlicherianum* (*MeATG3*). In addition, the three domains of ATG3 were more conserved within plants vs. animals. However, the key, functionally necessary Cys-234 residue was detected in the ATG3s of all species. Nineteen amino acids were highly conserved among plant species but differed from those of yeast and human/mouse.

We predicted the secondary structures of the ATG3s based on the crystal structure of ScATG3 (Additional file 1A). The Autophagy_N, Autophagy_act_C and Autophagy_C domains comprise three alpha helices (α1, α2, α3) and two beta sheets (β1, β2), two alpha helices (α4, α5) and three beta sheets (β4, β5, β6), and one alpha helix (α7), respectively. β3 is partially contained in the Autophagy_act_C domain, and α6 is positioned between the Autophagy_act_C and Autophagy_C domains. Eight motifs (1, 2, 3, 4, 5, 6, 8 and 10) are present ATG3 proteins from both plants and animals, while two motifs (7 and 9) are present only in plants (Additional file 1B). Sequence alignment and motif analysis pointed to the divergence of ATG3s between plants and animals. Phylogenetic analysis also showed that the *ATG3* genes were clustered into two different clades (Additional file 1C). These results indicate that these genes have undergone early divergence and independent evolution between the plant and animal lineages. In addition, the conserved characteristics of ATG3 between land plants and subaerial green algae suggest that the functional divergence of these genes occurred prior to land plant terrestrialization.

### Tissue-specific expression profiles and subcellular localization of PpATG3

**Fig. 1:**
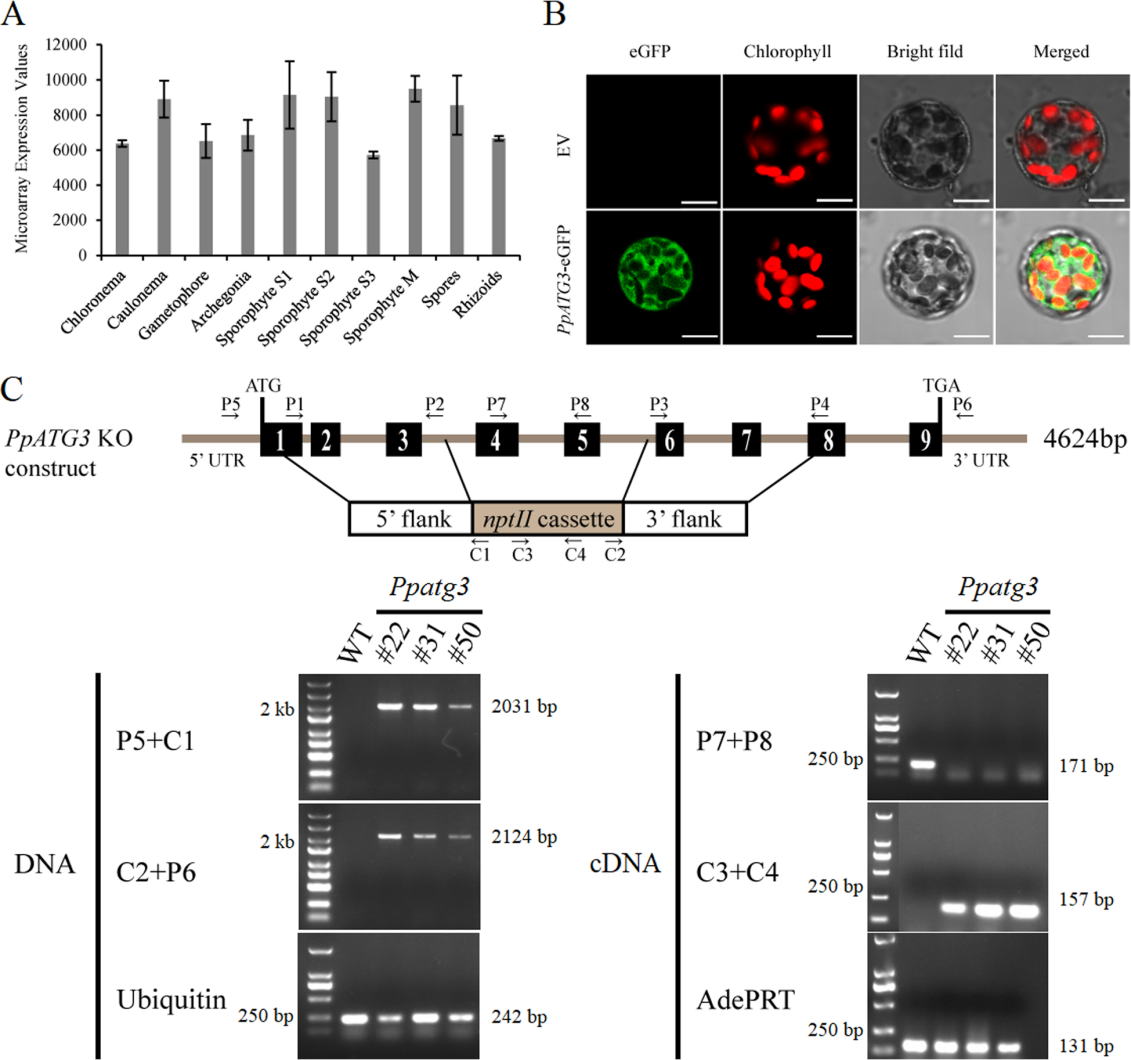
Tissue-expression profiles, subcellular localization and targeted disruption of *PpATG3* gene. **a** *PpATG3* expression profiles in different *P. patens* tissues. The expression data was retrieved from a previous research by Ortiz-Ramírez et al. **b** Subcellular localization of *PpATG3*. Confocal microscopy images of *P. patens* protoplasts by PEG-mediated transformation with empty vector (EV) or with *p35S:PpATG3-eGFP* construct. The scale bar = 10 μm. **c** Targeted disruption of *PpATG3* gene and PCR confirmation. Schematic representation showing deletion of Exons 4–5 that corresponds to removal of a 573 bp genomic region and insertion of a 2078 bp *nptII* cassette. Right and left arrows were indicated forward and reverse primers, respectively. PCR analysis was used to verify genomic insertion of *nptII* cassette and loss of *PpATG3* transcripts. Primer pairs of P5/C1 and C2/P6 were used for verifying double-ended insertion of the *nptII* cassette at genomic level. Primer pairs of P7/P8 and C3/C4 were used for verifying the loss of *PpATG3* transcripts and the expression of *nptII* cassette, respectively. *PpUbiquitin* and *PpAdePRT* were used as a DNA or cDNA template quality control, respectively. The fragment length and DNA size markers were shown on the gel right and left, respectively

To assess the expression patterns of *PpATG3* in different tissues, we retrieved the corresponding microarray data from the transcriptome of *P. patens* [32]. *PpATG3* was expressed at high levels throughout the *P. patens* life cycle, with transcript abundance (robust multi-array average) values > 5000 (Fig. 1a). To determine the subcellular localization of PpATG3 in *P. patens*, we fused the full-length coding sequence of *PpATG3* with that of enhanced green fluorescent protein (eGFP) in-frame under the control of the constitutive CaMV35S promoter (*p35S:PpATG3-eGFP*) and transiently transformed *P. patens* protoplasts with this construct (Fig. 1b). We used the empty vector (EV) as a control (Fig. 1b). Confocal microscopy revealed that the fluorescent signal of the *PpATG3*-eGFP fusion proteins was evenly distributed in the cytoplasm of the protoplasts, whereas the EV control did not generate a signal.

### *PpATG3* knockout disrupts gametophore senescence and protonema formation

**Fig. 2:**
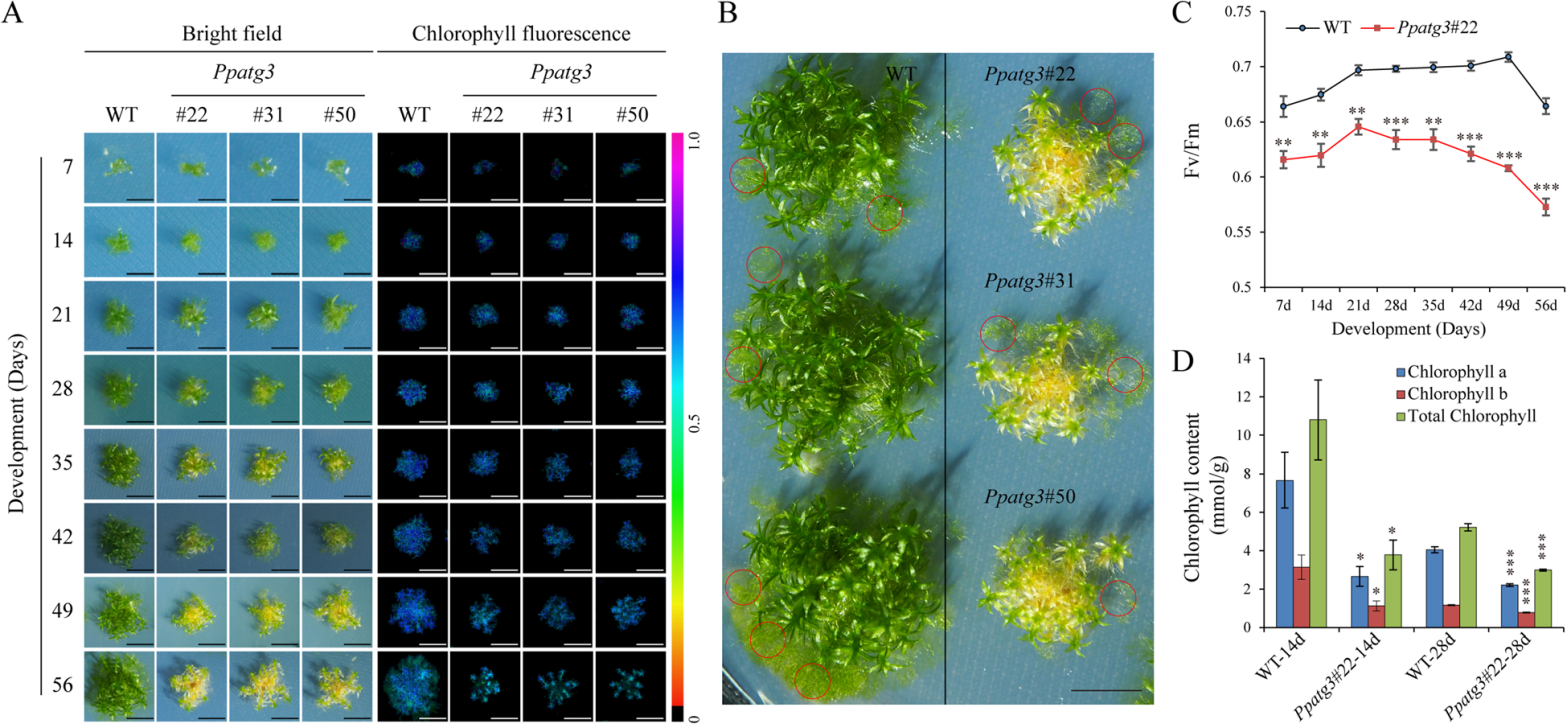
*PpATG3* affects growth and photosynthetic regulation in *P. patens*. **a** WT and *Ppatg3* knockout plants were observed after growing 7 to 56 days at normal growth conditions. The scale bar = 4 mm. **b** *PpATG3* affects the formation of new protonemata. The 56-day-old plants were used for analysis and the red circles were indicated newly formed protonemata. The scale bar = 4 mm. **c** Fv/Fm values of WT and *Ppatg3* plants. **d** Chlorophyll decreased in the *Ppatg3* knockout plants. Three biological replicates were analyzed and error bars show the mean value ± SD. The asterisks indicate a significant change between the *Ppatg3* and WT plants at (*) *p* < 0.05, (**) *p* < 0.01, and (***) *p* < 0.001

To further explore the role of *PpATG3*, we generated *Ppatg3* knockout transgenic plants by disrupting exons 4 and 5 through homologous recombination (HR) (Fig. 1c). This yielded three knockout lines (ko#22, ko#31 and ko#50) of *PpATG3*, whose identities were confirmed by PCR. We isolated genomic DNA and total RNA from these plants to verify the genomic insertion of the *nptII* cassette and loss of *PpATG3* transcripts due to HR events at its 5′ and 3′ flanks (Fig. 1c), respectively. *PpATG3* had been successfully disrupted at the genomic locus via the insertion of a 2078-bp *nptII* cassette into both arms of the target by HR. To investigate whether *PpATG3* functions in gametophore development in *P. patens*, we examined 7- to 56-day-old wild-type (WT) and *Ppatg3* knockout plants under normal growth conditions. There was a significant difference between *Ppatg3* knockout and WT plants, with the mutant showing an increasingly premature-senescence phenotype over time (Fig. 2a). The chlorophyll fluorescence of the *Ppatg3* mutant became weaker than that of WT plants (Fig. 2a). This premature senescence was most notable in 56-day-old plants, as the stem sections and basal leaves of leafy gametophores in the *Ppatg3* knockout plants turned yellow (Fig. 2b). In addition, in 56-old-day plants, there were far fewer newly formed protonemata in *Ppatg3* knockout plants compared to WT plants (Fig. 2b, red circles).

The photosynthetic yield (Fv/Fm) values also differed in 7-day-old *Ppatg3* knockout vs. WT plants, and subsequently the mutant showed seriously decreased fluorescence compared to WT plants (Fig. 2c). This finding is supported by the reduced chlorophyll biosynthesis in *Ppatg3* knockout plants: the chlorophyll a, chlorophyll b and total chlorophyll contents were significantly lower in both 14- and 28-day-old *Ppatg3* mutant vs. WT plants (Fig. 2d). However, the chlorophyll contents were also slightly lower in 28-day-old WT plants than in 14-day-old WT plants, likely because more protonemata were present in younger plants. These results indicate that the *Ppatg3* knockout plants underwent a greater reduction in chlorophyll content than the WT, resulting in an early-senescence phenotype.

### *PpATG3* dysfunction affects cell development in *P. patens*

**Fig. 3:**
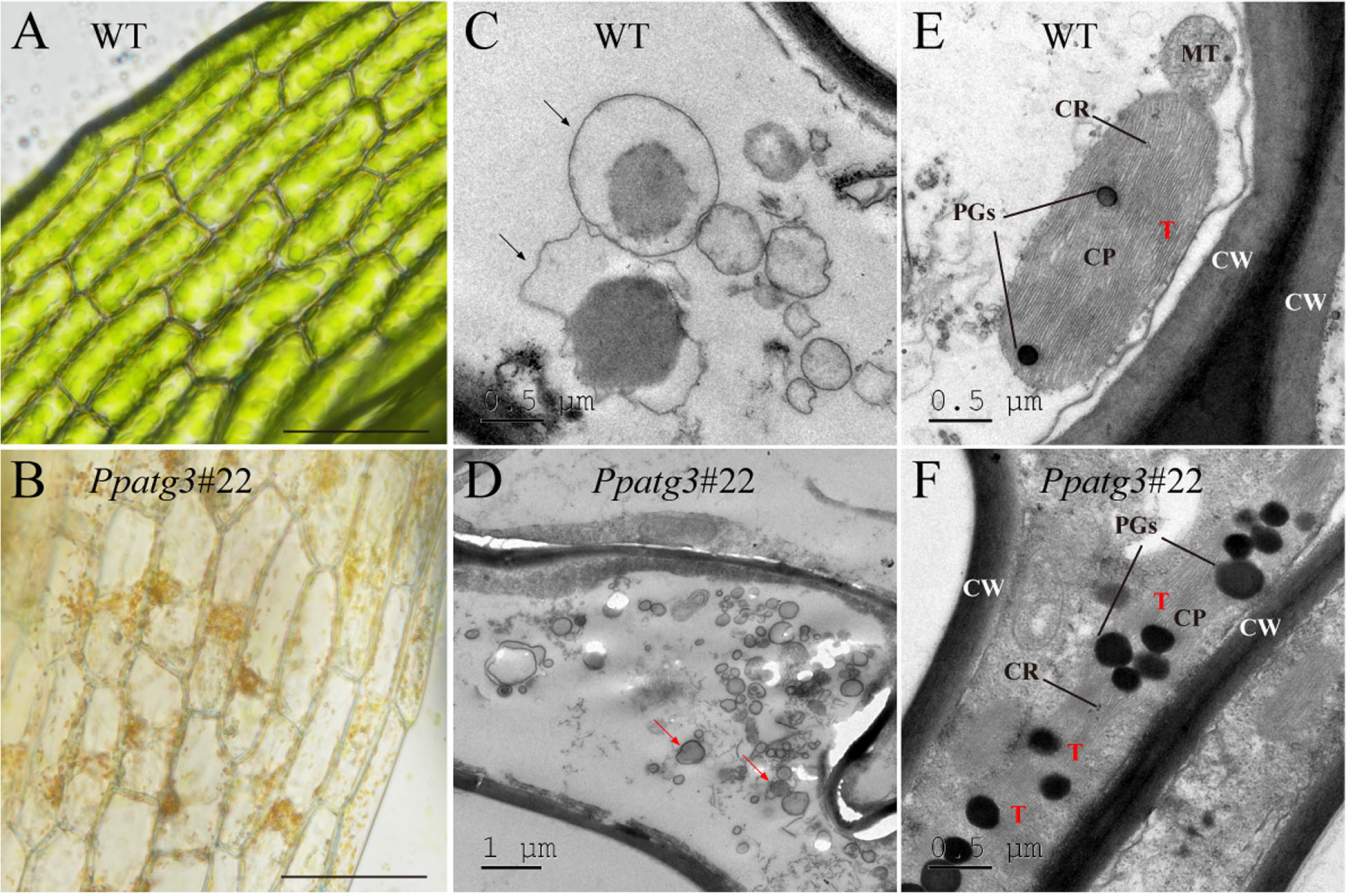
*PpATG3* affects the cell development in *P. patens*. **a** and **b** Leafy gametophore cells were observed by light microscopy. The scale bar = 0.3 mm. **c** and **d** Detection of autophagosome in the gametophore cells of WT and *Ppatg3* knockout plants by TEM. The 28-day-old plants after treatment for 1 h of 100 mM NaCl were used for analysis. The black arrows were indicated the formation of autophagosomes in WT and the red arrows were indicated the bulk cytosolic components accumulated in *Ppatg3* mutants due to autophagy defect. **e** and **f** *PpATG3* dysfunction causes the accumulation of chloroplast plastoglobuli. The 28-day-old plants at normal growth conditions were used for analysis. CP, chloroplast; PGs, plastoglobuli; T, thylakoid; CR, chloroplast ribosome; MT, mitochondrion; CW, cell wall

To explore how *PpATG3* regulates plant senescence, we examined the leafy gametophores cells of WT and *Ppatg3* plants grown under normal conditions in detail. The cells of the *Ppatg3* mutant appeared hollow and were turning yellow, whereas those of WT plants remained full and green (Fig. 3a and b). To validate that the deletion of *PpATG3* prevents autophagosome formation in *P. patens*, we treated 28-day-old WT and *Ppatg3* knockout plants with 100 mM NaCl for 1 h and observed them by transmission electron microscopy (TEM). Autophagosomes containing cellular cargos formed in WT plants (Fig. 3c), whereas the bulk cytosolic components accumulated in the mutant due to *PpATG3* knockout (Fig. 3d). These results indicate that autophagy was disrupted in the *Ppatg3* mutant.

To further explore the effect of *PpATG3* on senescence in moss, we examined chloroplasts in WT and *Ppatg3* cells. We observed a higher density of cellular substances in leafy gametophore cell from the *Ppatg3* mutant compared to WT (Fig. 3e and f). Moreover, in the mutant, these cells accumulated an unusually high density of chloroplast PGs; these lipoprotein particles play important roles in various metabolic processes such as photosynthetic regulation, thylakoid lipid remobilization and senescence [33]. The higher density of chloroplast PGs in *Ppatg3* leafy gametophore cells suggests that PGs accumulation might be related to the reduced chlorophyll levels in the autophagy mutant.

### Changes in C/N ratios and fatty acid contents

**Fig. 4:**
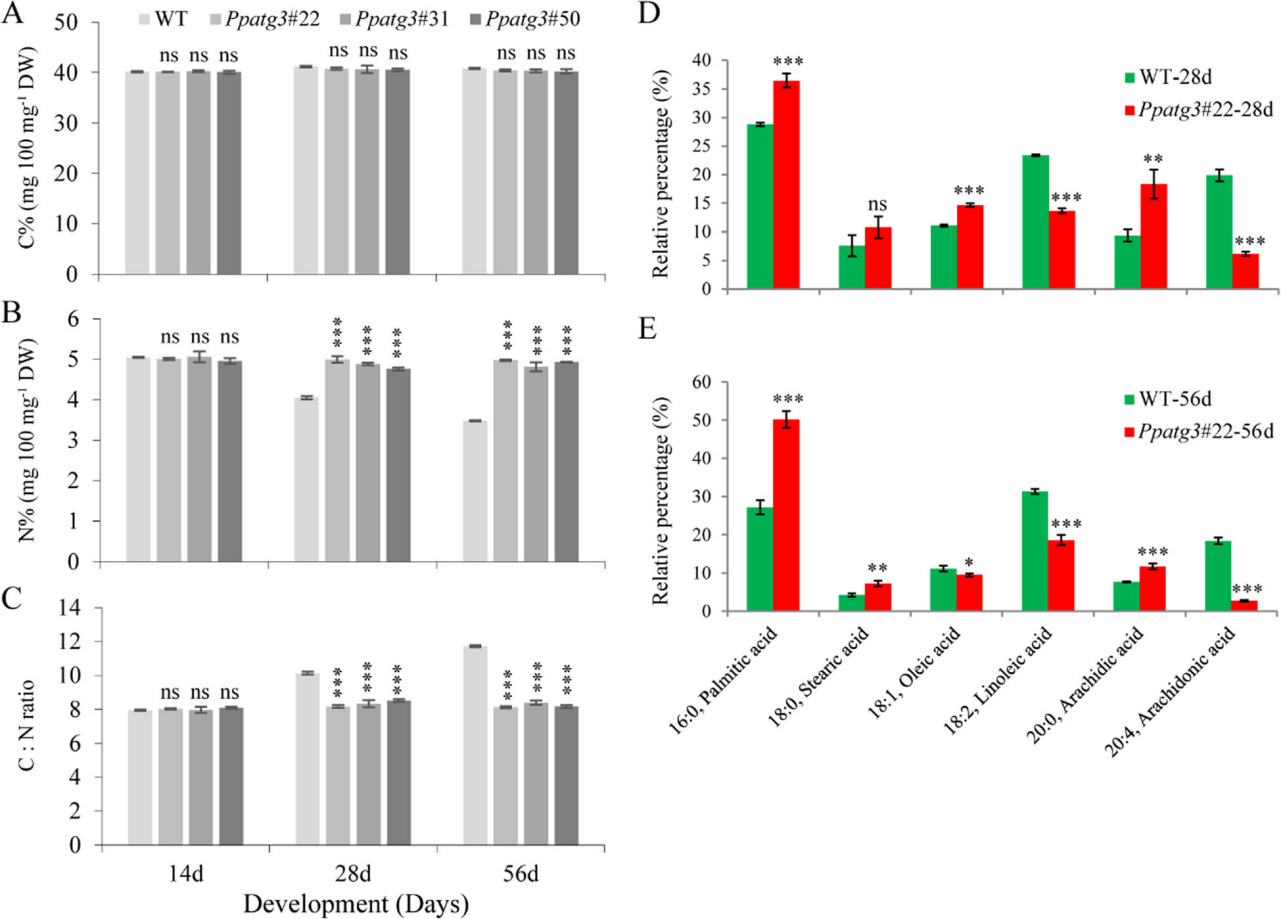
Comparison of C/N ratio and fatty acid content. **a**-**c** Differences in N concentrations between WT and *Ppatg3* resulted in changes in the C/N ratio. The 14-day-old, 28-day-old and 56-day-old plants were used for analysis. **d**-**e** Abundance comparison of six fatty acids from WT and *Ppatg3*. Fatty acid profiles were established from 28-day-old and 56-day-old plants. Three biological replicates were analyzed and error bars show the mean value ± SD. The asterisks indicate a significant change between the *Ppatg3* and WT plants at (*) *p* < 0.05, (**) *p* < 0.01, and (***) *p* < 0.001. Non-significant differences between the *Ppatg3* and WT plants are denoted (ns)

The C/N ratio is reduced in Arabidopsis autophagy mutant [29], and lipid metabolism is impaired in rice *OsATG7* knockout mutant [11]. Based on the hypothesis that changes in C/N ratios and fatty acid contents caused the early-senescence phenotype seen in *Ppatg3* knockout plants, we measured the C/N ratios of WT and *Ppatg3* plants at three time points: 14, 28 and 56 days (Fig. 4a–c). At 14 days, we did not detect any significant differences in C or N concentrations or C/N ratios between *Ppatg3* knockout and WT plants. At 28 and 56 days, however, *Ppatg3* plants showed notably lower C/N ratios than the WT due to higher N contents (N%). Overall, the N% rates gradually decreased over the three time points in WT plants, whereas they remained constant in *Ppatg3* knockout plants. These results suggest that N utilization was completely defective in the *Ppatg3* mutant. Notably, the C contents (C%) did not significantly differ between *Ppatg3* knockout and WT plants.

Beike et al. [34] detected high fatty acid (%) contents in the gametophores of wild-type *P. patens*. Here, we analyzed the contents of six fatty acids in *P. patens*: palmitic acid (16:0), stearic acid (18:0), oleic acid (18:1), linoleic acid (18:2), arachidic acid (20:0) and arachidonic acid (20:4). We chose two time points: 28 and 56 days (Fig. 4d–e). At 28 days, three fatty acids (palmitic acid, oleic acid and arachidic acid) showed significantly higher relative abundance (%) and two (linoleic acid and arachidonic acid) showed significantly lower relative abundance in the *Ppatg3* mutant compared to the WT. Similarly, at 56 days, three fatty acids (palmitic acid, stearic acid and arachidic acid) showed significantly higher and three (oleic acid, linoleic acid and arachidonic acid) showed significantly lower relative abundance in *Ppatg3* vs. the WT. By contrast, the relative stearic acid contents did not significantly differ between *Ppatg3* and WT plants at 28 days. Overall, the fatty acid profiles markedly differed between *Ppatg3* knockout and WT plants.

To further investigate the relationship between C/N ratio and fatty acid contents in the autophagy-defective mutant, we performed a fatty acid supplementation experiment. Because the linoleic acid and arachidonic acid contents were significantly reduced in the *Ppatg3* mutant (Fig. 4d–e), we hypothesized that these two fatty acids function in C/N status in *P. patens*. Indeed, supplementing WT plants with linoleic and arachidonic acids, either singly or together, altered the C/N status and decreased the C/N ratio compared to the control (Additional file 2A–C). By contrast, supplementing *Ppatg3* plants with linoleic acid or arachidonic acid alone did not improve N utilization, and supplementation with both linoleic acid and arachidonic acid reduced the N contents, resulting in a C/N ratio similar to that of WT plants (Additional file 2A–C). However, the premature gametophore senescence phenotype of the mutant was not rescued by fatty acid supplementation (Additional file 2D).

### RNA-Seq to identify differentially expressed genes in *Ppatg3*

To examine whether the loss of ATG3 affects the gene expression profile of *P. patens*, we analyzed the global gene expression pattern of the *Ppatg3* mutant compared to the WT control using the BGISEQ-500 platform. Transcripts with FPKM ≥1 were subjected to further analysis. PCA revealed highly significant transcriptional differences between *Ppatg3* and WT plants (Additional file 3 A). In total, 23,219/16,564 expressed transcripts/genes were detected from all samples, including 23,139/16,503 transcripts/genes expressed in both *Ppatg3* and WT plants, 45/38 unique transcripts/genes in *Ppatg3* and 35/23 unique transcripts/genes in WT (Additional file 3B and Additional file 4). Using the criteria of *p-*value ≤0.001 and expression fold change > 2 to identify differentially expressed transcripts/genes (DETs/DEGs), a comparison of *Ppatg3* and WT revealed a total of 3080/2634 DETs/DEGs. Of these, 1845/1621 DETs/DEGs and 1235/1013 DETs/DEGs were upregulated and downregulated, respectively, in *Ppatg3* vs. the WT (Additional file 5).

We then identified the top 20 enriched KEGG pathways of the up- and downregulated DETs/DEGs at *Q* value ≤0.05 (Additional file 3D–E and Additional file 6). Among both up- and downregulated DETs/DEGs, the enriched pathways were all biosynthetic and metabolic pathways, which were roughly divided into five major functional classes: carbohydrate metabolism, energy metabolism, amino acid metabolism, cofactor and vitamin metabolism, and global pathways. Notably, the nitrogen metabolism pathway was significantly enriched (Additional file 3 D), which might be related to the altered N contents of the *Ppatg3* mutant.

In Arabidopsis, the differential expression of senescence-associated genes (SAGs) and weakened photosynthetic capacity are associated with plant senescence [35]. Notably, numerous genes related to chlorophyll biosynthesis and photosystems were downregulated in *Ppatg3* vs. the WT (Additional file 7). In addition, half of the SAGs (11 of 22) were significantly upregulated in the *Ppatg3* mutant compared to the WT (Additional file 7). These results provide evidence for the accelerated senescence process in the *Ppatg3* mutant.

### The transcription of nitrogen and fatty acid/lipid metabolism-related genes is altered in the *Ppatg3* mutant

**Fig. 5:**
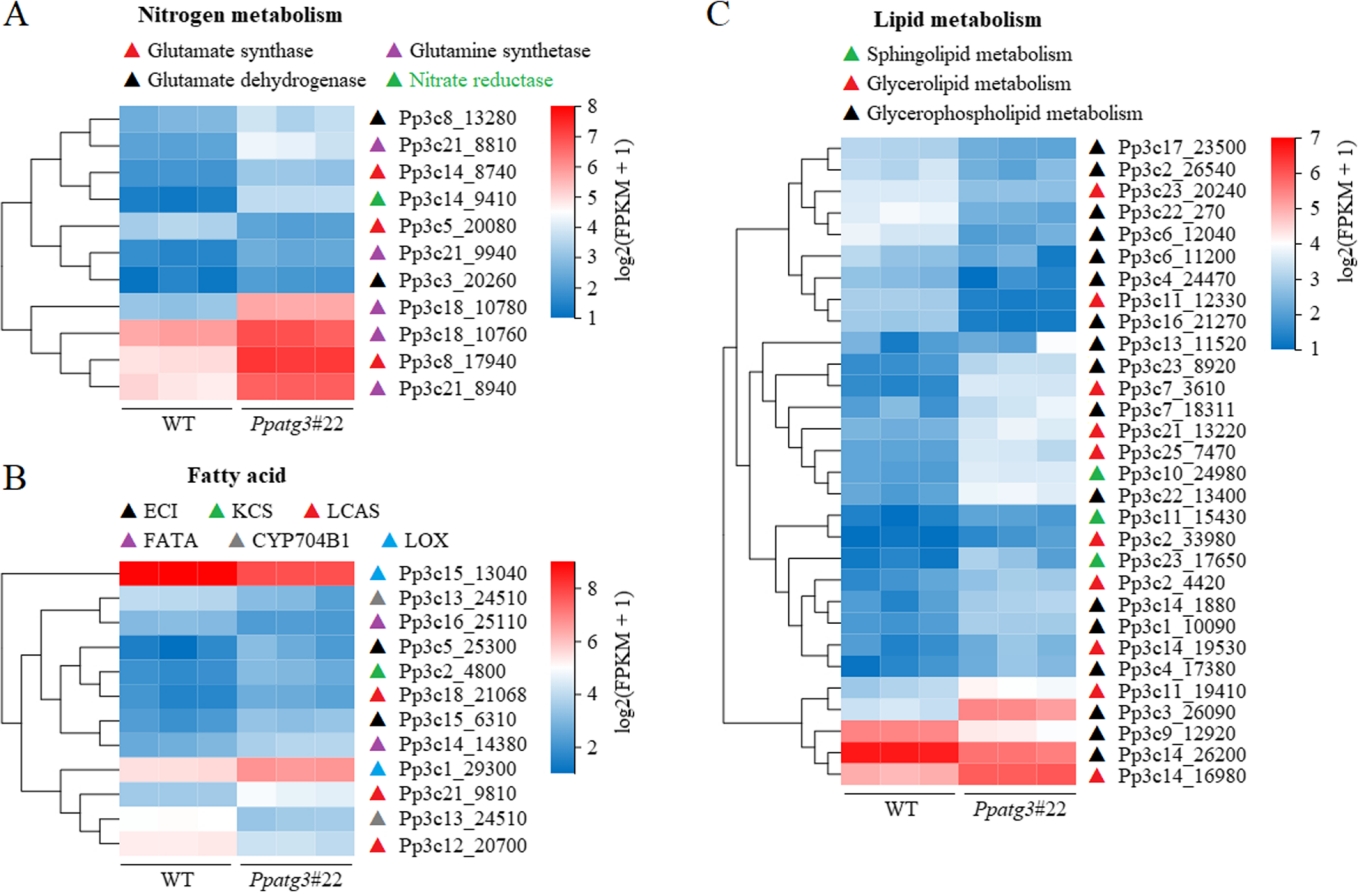
Differential expression of genes related to nitrogen metabolism and lipid/fatty acid metabolism in *Ppatg3* plants. **a**-**c** Transcriptional analysis for a subset of genes related to nitrogen metabolism and lipid/fatty acid metabolism in WT and *Ppatg3*. Expression levels shown as log2(FPKM+ 1) values. Three biological replicates were analyzed. Detailed information for each gene is supplied in Additional file 8

To further investigate the reason for the dysfunctional N and fatty acid metabolism in *Ppatg3*, we compared the differences in transcript levels of genes related to nitrogen and fatty acid/lipid metabolism. Ten of the 11 genes were significantly upregulated, including genes related to glutamine synthetase (GLN), glutamate synthase (GLS), nitrate reductase (NR) and glutamate dehydrogenase (GDH) (Fig. 5a and Additional file 8). These results indicate that the nitrogen metabolism pathway was defective in *Ppatg3*, resulting in the differential expression of genes involved in nitrogen metabolism. This phenomenon might be due to feedback regulation of nitrogen-related DEGs caused by a N-utilization deficiency in the *Ppatg3* mutant. However, the upregulated expression of these genes did not restore the N-utilization efficiency, suggesting that the regulation mechanism of autophagy for N utilization was more complicated.

Furthermore, 12 genes related to fatty acid biosynthesis and metabolism were significantly differentially expressed in the mutant, including 7 upregulated and 5 downregulated genes (Fig. 5b and Additional file 8). One upregulated gene, the lipoxygenase homologous gene (*LOX5*; Pp3c1_29300), might be involved in linoleic acid metabolism; its higher expression level is consistent with the reduced linoleic acid contents in *Ppatg3*. However, another lipoxygenase homologous gene (*LOX3*; Pp3c15_13040), which might be involved in the arachidonic acid metabolism, was downregulated in the mutant: its lower expression level might not be related to the reduced arachidonic acid contents in *Ppatg3*. Moreover, 19 of the 30 genes related to lipid metabolism were significantly upregulated in *Ppatg3*, including genes involved in glycerolipid, glycerophospholipid and sphingolipid metabolism (Fig. 5c and Additional file 8).

### Dysfunctional autophagy leads to the differential transcription of protein metabolism, endocytosis and ROS-related genes

**Fig. 6:**
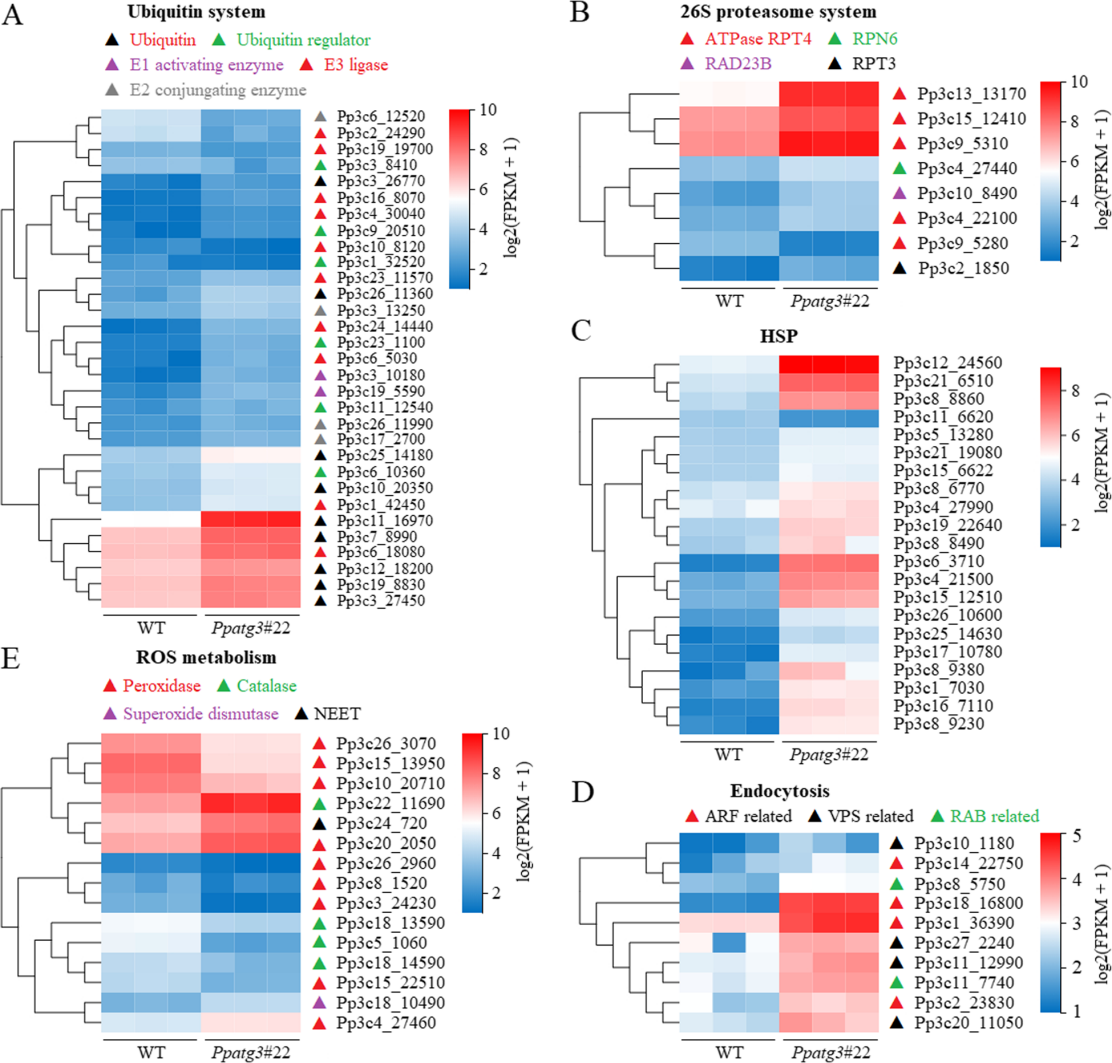
Transcriptional profiles of a subset genes related to protein metabolism, ROS metabolism and endocytosis in *Ppatg3* plants. **a**-**c** Differentially expressed genes related to ubiquitin, 26S proteasome and HSP, respectively. **d** Differentially expressed genes related to endocytosis. **e** Differentially expressed genes related to ROS metabolism. Expression levels shown as log2(FPKM+ 1) values. Three biological replicates were analyzed. Detailed information for each gene is supplied in Additional file 8

Twenty-five out of 31 ubiquitin-related genes were significantly upregulated in the *Ppatg3* mutant vs. the WT (Fig. 6a and Additional file 8). These highly expressed genes encode proteins including ubiquitin proteins or regulators, ubiquitin-activating enzymes (E1), ubiquitin-conjugating enzymes (E2) and ubiquitin ligases (E3). Moreover, the transcription of genes in the 26S proteasome system was activated by the upregulation of a subset of regulatory genes in the mutant (Fig. 6b and Additional file 8). These results suggest that the activity of the ubiquitin-26S proteasome pathway (UPP) for protein degradation is enhanced in the mutant due to a defect in autophagy.

Heat shock proteins (HSPs) play essential roles in preventing the misfolding of proteins and blocking the formation of large protein aggregates which severely impede cellular functions [36]. The transcript levels of many genes (20 of 21) encoding HSPs/chaperones were significantly higher in *Ppatg3* than the WT (Fig. 6c and Additional file 8), suggesting that the resolving of misfolded protein aggregates by HSPs/chaperones was activated in the mutant to maintain the proper protein conformation and extend cell longevity.

Furthermore, 10 genes related to the endocytosis pathway were significantly upregulated in the mutant (Fig. 6d and Additional file 8); this pathway is involved in the recruitment and degradation of cell surface proteins and cellular fatty acids/lipids to support basic cellular functions [37, 38]. However, in contrast to the highly expressed genes related to protein metabolism and endocytosis, the transcript levels of most ROS metabolism-related genes (10 of 15) were significantly reduced in the mutant, including 7 and 3 genes encoding peroxidases and catalases, respectively (Fig. 6e and Additional file 8), perhaps leading to the accumulation of ROS and the generation of damaged or toxic materials in the *Ppatg3* mutant.

### Validation of DEGs by RT-qPCR

**Fig. 7:**
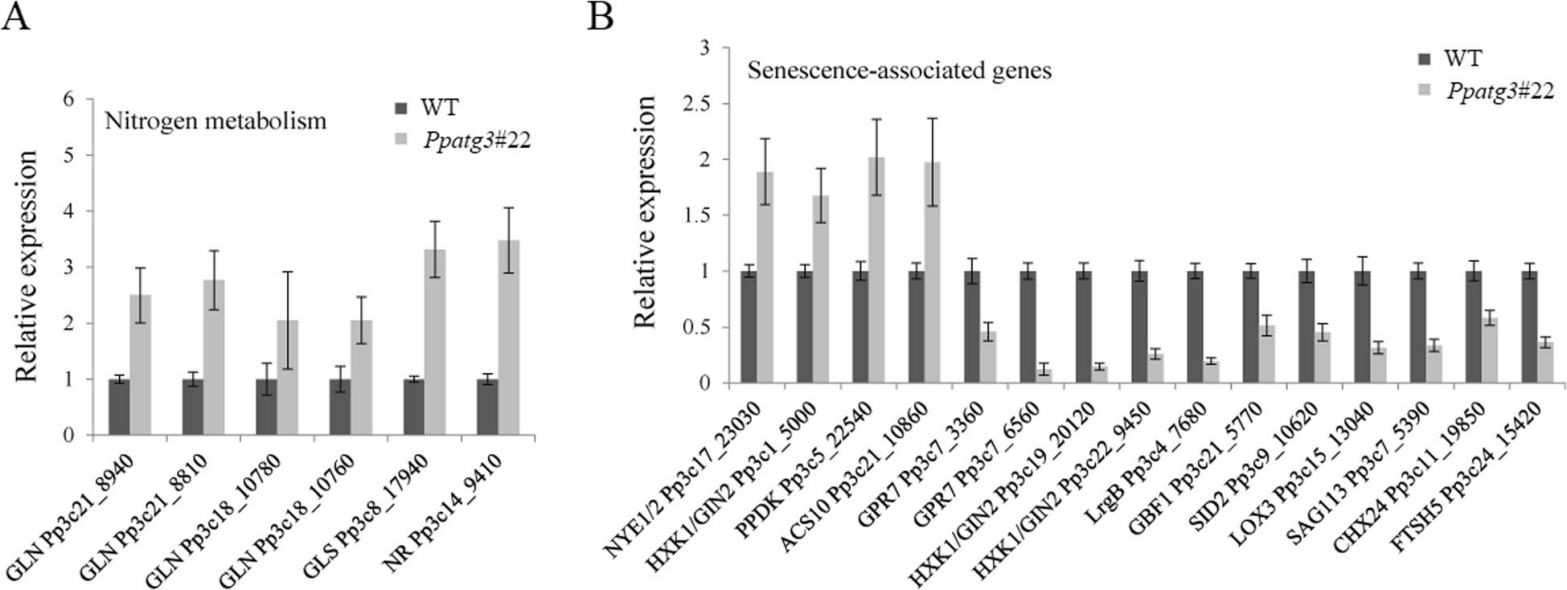
Transcript abundances of the nitrogen metabolism related genes (**a**) and SAGs (**b**) were confirmed by RT-qPCR. Three biological replicates were analyzed and error bars show the mean value ± SD. The expression value of WT sample was normalized to 1

Finally, to validate the gene expression patterns demonstrated by RNA-Seq, we performed RT-qPCR analysis of 21 DEGs, 15 homologous SAGs and 6 genes related to nitrogen metabolism using the same mRNA samples used for RNA-Seq analysis. These genes included several nitrogen metabolism-related genes, including homologs of *GLN* (Pp3c21_8940, Pp3c21_8810, and Pp3c18_10780 and Pp3c18_10760), *GLS* (Pp3c8_17940) and *NR* (Pp3c14_9410) (Fig. 7a). We also identified 4 and 11 SAG homologs that were up- and downregulated, respectively, in *Ppatg3* knockout plants (Fig. 7b), including homologs of *NYE1/2* (Pp3c17_23030), *HXK1/GIN2* (Pp3c1_5000, Pp3c19_20120, and Pp3c22_9450), *PPDK* (Pp3c5_22540), *ACS10* (Pp3c21_10860), *GPR7* (Pp3c7_3360 and Pp3c7_6560), *LrgB* (Pp3c4_7680), *GBF1* (Pp3c21_5770), *SID2* (Pp3c9_10620), *LOX3* (Pp3c15_13040), *SAG113* (Pp3c7_5390), *CHX24* (Pp3c11_19850) and *FTSH5* (Pp3c24_15420). The expression patterns of all genes examined were similar to those obtained by RNA-Seq analysis.

## Discussion

Autophagy is a ubiquitous process that plays important roles in plant development and senescence to maintain essential cellular functions and life activities [3, 7, 19]. Extensive studies have indicated that autophagy is important for N utilization [26–29], fatty acid/lipid homeostasis [13–15] and the degradation of damaged chloroplasts [16, 17] or aggregated proteins [18] in plants. Although a previous study revealed that autophagy is essential for maintaining the balance of amino acid metabolism in *P. patens* [30], how this process regulates C/N status and fatty acid metabolism in moss has been largely unknown. Here, we demonstrated that the E2-like enzyme PpATG3, which is extensively expressed in tissues (Fig. 1a) and is localized to the cytoplasm (Fig. 1b), is essential for both autophagy and normal plant development in *P. patens*. Thus, *Ppatg3* mutant cultured on normal growth medium for 7 to 56 days showed significantly premature senescence of leafy gametophores (Fig. 2a) and reduced new protonema formation (Fig. 2b) compared to WT plants grown under the same conditions.

Early leaf senescence is the principal phenotype of autophagy mutant in Arabidopsis [19, 39]. Thus, we examined several physiological and metabolic markers and performed transcriptome analysis of the *Ppatg3* mutant during the appearance of premature senescence in leafy gametophores. After 7 days of culture, yellowing and weak chlorophyll fluorescence were detected in *Ppatg3* (Fig. 2a), which is consistent with the significantly reduced Fv/Fm values of this mutant (Fig. 2c). After this time point, more serious yellowing was observed, indicating that the *Ppatg3* was indeed undergoing premature senescence. Indeed, the chlorophyll contents were significantly lower in the *Ppatg3* mutant than in WT plants (Fig. 2d). In addition, *Ppatg3* cells appeared hollow and yellow (Fig. 3a and b), which was accompanied by defects in autophagosome formation and led to the accumulation of bulk cytosolic cargos (Fig. 3c and d). These results suggest that physiological defects were present in this autophagy mutant, leading to a premature-senescence phenotype.

The physiological defects in *Ppatg3* plants were more severe later in the culture period, at 28 and 56 days. At 14 days, Fv/Fm values and chlorophyll biosynthesis were already significantly reduced in the mutant (Fig. 2c and d), but there was no significant difference in N content or C/N ratio between WT and *Ppatg3* plants (Fig. 4a–c). By contrast, at 28 days, the *Ppatg3* mutant not only exhibited reduced photosynthetic capacity (Fig. 2c and d), but they also displayed dysfunctional nitrogen metabolism resulting in a lower C/N ratio (Fig. 4a–c). An autophagy-defective Arabidopsis mutant displays reduced N levels, resulting in a higher C/N ratio [29]. By contrast, the lower C/N ratio in the *Ppatg3* mutant relative to WT plants was clearly linked to dysfunctional nitrogen metabolism at both 28 and 56 days (Fig. 4a–c). These results indicate that the effects of autophagy on nitrogen metabolism differ between *P. patens* and *A. thaliana*.

Chloroplast PGs are lipoprotein particles with a high lipid-to-protein ratio that function in chloroplast biogenesis whose numbers increase during the process of plant senescence [33]. Estimates suggest that 75–80% of the total nitrogen content in a plant leaf is stored in chloroplasts [40]. In the current study, we observed irregular chloroplasts with a high density of PGs in leafy gametophore cells of *Ppatg3* but not WT plants (Fig. 3e–f). The defects in chlorophyll biosynthesis and N metabolism in the mutant might have contributed to its defective chloroplast development. Chloroplasts with accumulated PGs in Arabidopsis were regular during normal senescence [33]; however, the chloroplasts that accumulated PGs in *Ppatg3* appeared damaged, with an irregular shape. Perhaps a defect in autophagy in the *Ppatg3* mutant impaired the degradation and turnover process of unnecessary or damaged chloroplasts, leading to the accumulation of PGs and abnormally shaped chloroplasts, thereby resulting in reduced chlorophyll levels.

Autophagy plays important roles in lipid/fatty acid metabolism, composition and turnover in several vascular plants, such as Arabidopsis and maize [13, 14]. Moreover, in rice, the metabolism of lipids such as triacylglycerols (TAGs) and diacylglycerols (DAGs) was impaired during pollen maturation in an autophagy mutant [11]. Fatty acids are important components of TAGs and DAGs [41]; however, the potential role of autophagy in fatty acid metabolism in moss has not been reported. In the current study, we discovered that the loss of autophagy affected the relative levels of six fatty acids, resulting in their impaired metabolism (Fig. 4d–e). In particular, the relative levels of linoleic acid (18:2) and arachidonic acid (20:4) were sharply reduced in the *Ppatg3* mutant. Supplementation with linoleic acid and arachidonic acid together, but not individually, restored the C/N ratio of the mutant compared to the WT (Additional file 2A–C), suggesting that the disrupted N metabolism in the mutant might be related to the reduction in fatty acid contents. These results suggest that the early-senescence phenotype of *Ppatg3* was partially caused by impaired N utilization and an imbalance in fatty acid contents. However, the premature gametophore senescence was not relieved by fatty acid supplementation (Additional file 2D), suggesting that autophagy-dependent senescence is influenced by other factors besides the C/N ratio and fatty acid metabolism, an issue worthy of further study.

During the normal senescence process, fatty acids accumulate in chloroplast PGs and chlorophylls and lipids are extensively degraded [42]. In rice, senescence is accompanied by an increase in the number of chloroplast PGs and significant changes in the contents of fatty acids, especially increases in palmitic acid (16:0) levels [43]. Similarly, in the current study, we observed that the *Ppatg3* mutant contained an unusually high density of chloroplast PGs (Fig. 3e and f) and high palmitic acid (16:0) contents (Fig. 4d and e), which is consistent with previous reports. In addition, the *Ppatg3* mutant contained significantly higher levels of another fatty acid, arachidic acid (20:0), compared to the WT in both 28- and 56-day-old plants (Fig. 4d and e). However, the levels of two fatty acids, stearic acid (18:0) and oleic acid (18:1), behaved differently at 28 vs. 56 days (Fig. 4d and e). These results suggest that changes in fatty acid contents during autophagy-dependent senescence might also be related to PG accumulation in chloroplasts, which might have partially contributed to the physiological defects of the autophagy mutant.

Additionally, RNA-Seq revealed that the loss of ATG3 in *P. patens* significantly altered transcript abundance/gene expression (Additional file 3). Analysis of the top 20 enriched KEGG pathways of the DEGs suggested that biosynthetic and metabolic pathways were seriously affected in the mutant (Additional file 3D–E and Additional file 6). As expected, the nitrogen metabolism pathway was significantly enriched (Additional file 3D) by the differential expression of a subset of nitrogen-related genes (Fig. 5a), which might be related to the N contents of the *Ppatg3* mutant. Furthermore, many genes involved in fatty acid/lipid metabolism were significantly differentially expressed in the mutant vs. the WT (Fig. 5b and c), which might be associated with the changes in fatty acid contents and premature senescence due to defective autophagy. Dysfunctional autophagy led to the increased transcription of genes associated with UPP and HSP, a phenomenon that also occurs in endocytosis-related genes (Fig. 6a–d). These findings suggest that the degradation of unnecessary proteins or fatty acids/lipids is promoted in the *Ppatg3* mutant for recycling to support basic cellular functions and extend longevity. However, a subset of ROS metabolism-related genes was significantly downregulated in the mutant (Fig. 6e and Additional file 8), which might have contributed to senescence resulting from damaged or toxic materials generated by ROS accumulation in *Ppatg3*.

As described in Arabidopsis [35], numerous genes related to SAGs, chlorophyll biosynthesis and photosystems were differentially expressed in *Ppatg3* vs. the WT plants, suggesting that the senescence process began early in the *Ppatg3* mutant (Additional file 7). For example, the *P. patens* homologs of *NYE1/2* and *PPDK* were upregulated in the *Ppatg3* mutant, as evidenced by both RNA-Seq analysis and RT-qPCR validation. *NYE1/2* and *PPDK* encode important regulators of chlorophyll degradation and nitrogen remobilization, respectively, that function during green leaf senescence in Arabidopsis [44–46] (Additional file 7 and Fig. 7b). These results suggest that the chlorophyll degradation and nitrogen utilization mechanisms might be conserved to some degree between autophagy-defective senescence and normal green leaf senescence. However, several *P. patens* SAG homologs, such as *SAG113*, were downregulated in the *Ppatg3* mutant (Additional file 7 and Fig. 7b), pointing to possible differences in the regulatory mechanisms of autophagy-defective senescence vs. normal green leaf senescence.

A recent study in yeast revealed that the ATG3 allosteric activation switch element E123IR plays essential roles in removing intramolecular interactions of ATG3 with the E1-like enzyme ATG7 and E3-like enzyme ATG12-ATG5 complex to remodel the active site of ATG3, thereby mediating the lipidation reaction of ATG8–PE during the autophagy process [47]. As expected, Y2H assays demonstrated that PpATG3 strongly interacts with PpATG7 and PpATG12 in *P. patens* (Additional file 9), suggesting that the interaction network of ATG3 with ATG7 and ATG12 might be conserved. However, whether the exact interaction mechanism of ATG3 with E1- or E3-like enzymes in plants is identical to that discovered in yeast requires further study.

Taken together, these results indicate that the premature-senescence phenotype caused by *Ppatg3* knockout is influenced by a series of complex metabolic pathways, including N utilization, fatty acid/lipid metabolism, damaged chloroplast degradation, ROS removal and the recycling of unnecessary proteins due to the disruption of autophagy. However, the exact mechanisms underlying the roles of autophagy in these physiological defects should be more fully elucidated in the future.

## Conclusions

Our analysis of *P. patens*, an emerging model system for autophagy research, provided new insights into the role of autophagy in preventing plant senescence. Our results support the role of *PpATG3* in autophagosome formation. Autophagy-defective mutant due to a *PpATG3* deletion showed premature gametophore senescence under non-stress conditions, which could be partially explained by impaired N metabolism. We also provided evidence for the role of autophagy in fatty acid metabolism. The *Ppatg3* mutant showed reduced levels of linoleic acid and arachidonic acid and a concomitant increase in the levels of palmitic acid and arachidic acid. Supplementation with linoleic acid and arachidonic acid together restored the N content but not the premature senescence of the mutant, suggesting that a more complex mechanisms contributes to autophagy-dependent longevity. Our results also suggest that the differential expression of numerous biosynthetic and metabolic pathway genes played a role in the premature senescence of the *Ppatg3* mutant due to defective autophagy. These results suggest that *PpATG3* directly or indirectly affects C/N stability and fatty acid metabolism, as well as numerous other biosynthetic and metabolic pathways, such as damaged chloroplast degradation and unnecessary protein recycling, by functioning in autophagy under normal growth conditions, thus preventing premature senescence and extending plant longevity.

## Methods

### Plant material, growth condition and moss transformation

*Physcomitrella patens* Gransden wild-type plants were supplied by Prof. Mitsuyasu Hasebe, Japan National Institute for Basic Biology (NIBB). Plants were grown on BCD medium supplemented with 5 mM ammonium tartrate and 1 mM CaCl_2_ [48] at 25 °C under a photoperiod of 16 h light with a photon flux of 60 to 80 μmol m^− 2^ s^− 1^ for 1 to 8 weeks. To generate protonemal tissues, plant material was crushed with an ULTRA-TURRAX® Tube Drive in 5 ml of sterile water and was propagated onto BCD medium. Cultures were maintained by inoculating a small explant of seven-day-old protonemal tissue (1–2 mm) using forceps to fresh culture medium. Polyethylene glycol (PEG)-mediated transformation of protoplasts for gene transient expression or gene stable deletion was described previously by Shi and Theg [49].

### Protein sequence alignment, motif and phylogenetic analysis

The full-length protein sequences of the *PpATG3* (Pp3c8_11900), *KnATG3* (GAQ83284), *MeATG3* (ME000344S06021), *AaATG3* (AANG000634), *MpATG3* (Mapoly0003s0208), *BdATG3* (Bradi3g33350) *AtATG3* (AT5G61500), *MmATG3* (NP_080678), *HsATG3* (NP_071933) and *ScATG3* (YNR007C) were obtained from Phytozome, NCBI and SGD database, respectively. ALN file was obtained by multiple protein sequences alignment using ClustalX2.0 [50], and the crystal structure of ScATG3 (PDB ID: 2DYT) [21] was used for secondary structure depiction by web tool ESPript 3.0 [51]. The domain and motif searching were performed by SMART [52] and MEME SUITE [53], respectively. The phylogenetic tree was carried out by MEGA 6 [54] using a neighbour-joining (NJ) method.

### Microarray expression analysis

Microarray expression values in different tissues of PpATG3 gene was analyzed basing previous microarray data [32] of *P. patens*. Ten tissues were obtained for analysis including protonemata (chloronema and caulonema), gametophore, archegonia, four sporophyte developing stages (S1, S2, S3 and M), spores, and rooting structure rhizoids.

### Subcellular localization of PpATG3

Full length coding sequence (lacking the stop codon) of *PpATG3* was PCR amplified from *P. patens* cDNA template using the primers PpATG3-F and PpATG3-R (Additional file 10). The resulting 921-bp PCR fragment was cloned using *KpnI* and *XbaI* restriction enzymes (Thermo Scientific FastDigest, FD0524 and FD0684) into the vector pM999 [55] by sticky end ligation. The pM999 contains the eGFP coding sequence driven by a 35S promoter. The chimeric gene *p35S:PpATG3-eGFP* was constructed by fusing the *PpATG3* cDNA sequence to the 5′ end frame of eGFP. After this, the resulting plasmid was introduced into the protoplast of *P. patens* by PEG-mediated transformation [48]. 48 h after culture at 25 °C, protoplasts were observed by a confocal microscopy for GFP fluorescence.

### *PpATG3* gene disruption

The 5′ and 3′ flanking regions (863 bp and 800 bp respectively) of the *PpATG3* gene were amplified using 2 primer pairs P1/P2 and P3/P4, respectively. Two fragments were digested with KpnI/HindIII and XbaI/BamHI respectively (Thermo Scientific FastDigest, FD0524, FD0504, FD0684 and FD0054), and cloned by sequential into the pTN182 vector upstream and downstream of the *nptII* gene driven by a Pm35S promoter (geneticin resistance gene cassettes) (http://moss.nibb.ac.jp/). Construct was linearized with *KpnI* and *BamHI* prior to transformation. Transformation was completed as described previously [48]. The primers for genotyping the *PpATG3* gene knockout were used can be found in Additional file 10. *PpUbiquitin* [56] and *PpAdePRT* [57] was used to assess DNA and cDNA template quality, respectively.

### Analysis of chlorophyll fluorescence and chlorophyll content

Chlorophyll florescence values were monitored with an IMAGING-PAM FluorImager and Imaging Win software. Prior to determine Fv/Fm, the cultures were dark adaptation for minimum 30 min. Chlorophyll were isolated from cultures using DMF (N, N-dimethylformamide) as described previously [58]. Absorbance was measured at 647 and 664 of the supernatants using DMF as blank. Total chlorophyll content (chlorophyll a + chlorophyll b) was calculated using the formula Chlorophyll a (mmol/g) = [(12 × A664–3.11 × A647) × mL DMF] / mg Dry Weight and Chlorophyll b (mmol/g) = [(20.78 × A647–4.88 × A664) × mL DMF] / mg Dry Weight.

### Phenotypic observation

For recording phenotypic characterization of WT and *Ppatg3* plants, cultures were grown for 7 to 56 days under normal conditions. Leafy gametophores were observed by light microscopy. TEM was used to observe the *Ppatg3* mutant. Gametophores of 28-day-old WT and *Ppatg3* plants were fixed overnight in 3% glutaraldehyde and then postfixed for 2 h in 1% osmium tetroxide (OsO4), dehydrated in a serial ethanol gradient and embedded in Epon 812 resin. Samples were fixed at 4 °C. Serial and uniform-thickness of sections about 70 nm of gametophores leafy cells were generated by a Leica EM UC7 ultramicrotome. Sections were carried onto 100-mesh Cu grids, and then stained sequentially by 2% uranyl acetate solution and lead citrate. Afterward, the sections were observed in microscope JEM 1400Plus at 120,000 V. To detect autophagosomes of WT and *Ppatg3* mutant, 28-day-old plants were treated by 100 mM NaCl for 1 h and then used for TEM observation.

### Measurement of carbon and nitrogen content

Plants were collected and dried 10 h at 100 °C in a drying oven. Then the subsamples were ground into a homogenous fine powder and carefully measured in tin capsules by a fully automatic C, N analyzer Elementar vario Micro cube to establish carbon and nitrogen content.

### Fatty acid content analysis

In this study, 1 g moss tissue of WT and *Ppatg3* plants were used for lipid extraction. Then the samples were utilized for methyl esterification and detailed method was performed as previously described [34]. Fatty acid contents were measured by a GC-MS analyzer Agilent Technologies 7890A GC system. Peak identification and fatty acid consideration were performed with the MSD ChemStation software.

### Fatty acid supplementation

For fatty acid supplementation, 21-day-old WT and *Ppatg3* plants grown on normal BCD medium were transferred to BCD medium with 20 μg/ml linoleic acid (Shanghai yuanye, DC14635400) or 20 μg/ml arachidonic acid (Shanghai yuanye, B20540) for 7 days, or both with linoleic acid and arachidonic acid at the same concentration for 7 days. As a control, 21-day-old WT and *Ppatg3* plants grown on normal BCD medium were transferred to BCD medium with 0.1% DMSO for 7 days.

### RNA-sequencing and data analysis

WT and *Ppatg3* plants were grown on BCD medium for 28d. Total RNA of the whole plant was extracted by using a RnaExTM solution (Generay). cDNA library construction, sequencing and data analysis were carried out by the BGISEQ-500 platform established by BGI-Tech (Wuhan, China). The reads alignment and expression level calculation were conducted by using Bowtie 2 [59] and RSEM [60], respectively. A differential expression transcript/gene with at least two-fold change and *p*-value ≤0.001 was used as further analysis [61]. R program “princomp”, “phyper” and “pheatmap” were used to conduct PCA analysis, KEGG pathway enrichment and construction of heat maps, respectively. The RNA-Seq data of the present study had been deposited at the BIG genome sequence archive (GSA) under Bioproject identifier PRJCA001964 with accession number: SAMC116811 to SAMC116814.

### RNA isolation and real-time quantitative PCR

RnaExTM solution (Generay) was used for total RNA extraction. First-strand cDNA was synthesized by a reverse transcription kit (Transgen). Real-Time quantitative PCR (RT-qPCR) was performed as previously described [62]. The primers for gene expression analysis were used in this study can be found in Additional file 10. The relative expression levels were calculated using *PpAdePRT* [57] as expression control.

### Y2H analysis

To determine the protein interactions between PpATG3 and PpATG7 (Pp3c24_8100) or PpATG12 (Pp3c4_29920), we performed the Y2H assays (Clontech). In our study, the CDS of PpATG3 gene was amplified and cloned into bait vector pGBKT7 (BD), while the CDS of PpATG7 or PpATG12 gene was amplified and cloned into prey vector pGADT7 (AD). The Y2H assays were carried out by a Frozen-EZ Yeast Transformation II Kit (Zymo Research). The primers for Y2H assays in this study were listed in Additional file 10.

## Supplementary information

**Additional file 1.**

**Additional file 2.**

**Additional file 3.**

**Additional file 4.**

**Additional file 5.**

**Additional file 6.**

**Additional file 7.**

**Additional file 8.**

**Additional file 9 Placeholder Text**

**Additional file 10.**

**Additional file 11.**

**Additional file 12.**

## Data Availability

All data generated or analyzed in this study are included in this article and the additional files. Original files for figures can be found in Additional files 11 and 12. Other data are available from the corresponding author on reasonable request. The raw RNA-Seq data were submitted to the GSA database with the bioproject ID: PRJCA001964, with accession number SAMC116811 to SAMC116814 (https://bigd.big.ac.cn/gsa/).

## Abbreviations

*ATG*: *AuTophaGy*
Cys: Cysteine
SAGs: Senescence-associated genes
WT: Wild-type
PGs: Plastoglobuli
ROS: Reactive oxygen species
UPP: Ubiquitin-26S proteasome pathway
DETs: Differentially expressed transcripts
DEGs: Differentially expressed genes
FPKM: Fragments per kilobase of transcript per million fragments mapped
KEGG: Kyoto encyclopedia of genes and genomes
TEM: Transmission electron microscopy
RT-qPCR: Real-Time quantitative PCR

## References

[1] Yang Z, Klionsky DJ (2010). Eaten alive: a history of macroautophagy. Nat Cell Biol.

[2] Avila-Ospina L, Moison M, Yoshimoto K, Masclaux-Daubresse C (2014). Autophagy, plant senescence, and nutrient recycling. J Exp Bot.

[3] Üstün S, Hafrén A, Hofius D (2017). Autophagy as a mediator of life and death in plants. Curr Opin Plant Biol.

[4] Masclaux-Daubresse C, Chen Q, Havé M (2017). Regulation of nutrient recycling via autophagy. Curr Opin Plant Biol.

[5] Liao CY, Bassham DC (2020). Combating stress: the interplay between hormone signaling and autophagy in plants. J Exp Bot.

[6] Wang P, Mugume Y, Bassham DC (2018). New advances in autophagy in plants: regulation, selectivity and function. Semin Cell Dev Biol.

[7] Bassham DC, Laporte M, Marty F (2006). Autophagy in development and stress responses of plants. Autophagy..

[8] Xie Z, Klionsky DJ (2007). Autophagosome formation: core machinery and adaptations. Nat Cell Biol.

[9] Liu Y, Xiong Y, Bassham DC (2009). Autophagy is required for tolerance of drought and salt stress in plants. Autophagy..

[10] Daudi A, Cheng Z, O'Brien JA (2012). The apoplastic oxidative burst peroxidase in *Arabidopsis* is a major component of pattern-triggered immunity. Plant Cell.

[11] Kurusu T, Koyano T, Hanamata S (2014). *OsATG7* is required for autophagy-dependent lipid metabolism in rice postmeiotic anther development. Autophagy..

[12] Wang Y, Yu B, Zhao J (2013). Autophagy contributes to leaf starch degradation. Plant Cell.

[13] Havé M, Luo J, Tellier F (2019). Proteomic and lipidomic analyses of the *Arabidopsis atg5* autophagy mutant reveal major changes in endoplasmic reticulum and peroxisome metabolisms and in lipid composition. New Phytol.

[14] McLoughlin F, Augustine RC, Marshall RS (2018). Maize multi-omics reveal roles for autophagic recycling in proteome remodelling and lipid turnover. Nat Plants.

[15] Masclaux-Daubresse C, d'Andrea S, Bouchez I, Cacas JL (2020). Reserve lipids and plant autophagy. J Exp Bot.

[16] Otegui MS (2018). Vacuolar degradation of chloroplast components: autophagy and beyond. J Exp Bot.

[17] Woodson JD (2019). Chloroplast stress signals: regulation of cellular degradation and chloroplast turnover. Curr Opin Plant Biol.

[18] Yu L, Chen Y, Tooze SA (2018). Autophagy pathway: cellular and molecular mechanisms. Autophagy..

[19] Hanaoka H, Noda T, Shirano Y (2002). Leaf senescence and starvation-induced chlorosis are accelerated by the disruption of an *Arabidopsis* autophagy gene. Plant Physiol.

[20] Ichimura Y, Kirisako T, Takao T (2000). A ubiquitin-like system mediates protein lipidation. Nature..

[21] Yamada Y, Suzuki NN, Hanada T (2007). The crystal structure of *Atg3*, an autophagy-related ubiquitin carrier protein (E2) enzyme that mediates *Atg8* lipidation. J Biol Chem.

[22] Sakoh-Nakatogawa M, Kirisako H, Nakatogawa H, Ohsumi Y (2015). Localization of *Atg3* to autophagy-related membranes and its enhancement by the *Atg8*-family interacting motif to promote expansion of the membranes. FEBS Lett.

[23] Besteiro S, Brooks CF, Striepen B, Dubremetz JF (2011). Autophagy protein *Atg3* is essential for maintaining mitochondrial integrity and for normal intracellular development of *Toxoplasma gondii* tachyzoites. PLoS Pathog.

[24] Han S, Wang Y, Zheng X (2015). Cytoplastic glyceraldehyde-3-phosphate dehydrogenases interact with *ATG3* to negatively regulate autophagy and immunity in *Nicotiana benthamiana*. Plant Cell.

[25] Liu Y, Bassham DC (2012). Autophagy: pathways for self-eating in plant cells. Annu Rev Plant Biol.

[26] Guiboileau A, Yoshimoto K, Soulay F, Bataillé MP, Avice JC, Masclaux-Daubresse C (2012). Autophagy machinery controls nitrogen remobilization at the whole-plant level under both limiting and ample nitrate conditions in *Arabidopsis*. New Phytol.

[27] Chen Q, Shinozaki D, Luo J (2019). Autophagy and nutrients management in plants. Cells..

[28] Marshall RS, Vierstra RD (2018). Autophagy: the master of bulk and selective recycling. Annu Rev Plant Biol.

[29] Guiboileau A, Avila-Ospina L, Yoshimoto K (2013). Physiological and metabolic consequences of autophagy deficiency for the management of nitrogen and protein resources in *Arabidopsis* leaves depending on nitrate availability. New Phytol.

[30] Mukae K, Inoue Y, Moriyasu Y (2015). *ATG5*-knockout mutants of *Physcomitrella* provide a platform for analyzing the involvement of autophagy in senescence processes in plant cells. Plant Signal Behav.

[31] Sanchez-Vera V, Kenchappa CS, Landberg K (2017). Autophagy is required for gamete differentiation in the moss *Physcomitrella patens*. Autophagy..

[32] Ortiz-Ramírez C, Hernandez-Coronado M, Thamm A (2016). A transcriptome atlas of *Physcomitrella patens* provides insights into the evolution and development of land plants. Mol Plant.

[33] van Wijk KJ, Kessler F (2017). Plastoglobuli: plastid microcompartments with integrated functions in metabolism, plastid developmental transitions, and environmental adaptation. Annu Rev Plant Biol.

[34] Beike AK, Jaeger C, Zink F, Decker EL, Reski R (2014). High contents of very long-chain polyunsaturated fatty acids in different moss species. Plant Cell Rep.

[35] Wang X, Gao J, Gao S, Song Y, Yang Z, Kuai B (2019). The H3K27me3 demethylase *REF6* promotes leaf senescence through directly activating major senescence regulatory and functional genes in *Arabidopsis*. PLoS Genet.

[36] Jacob P, Hirt H, Bendahmane A (2017). The heat-shock protein/chaperone network and multiple stress resistance. Plant Biotechnol J.

[37] Fan L, Li R, Pan J, Ding Z, Lin J (2015). Endocytosis and its regulation in plants. Trends Plant Sci.

[38] Jaishy B, Abel ED (2016). Lipids, lysosomes, and autophagy. J Lipid Res.

[39] Doelling JH, Walker JM, Friedman EM, Thompson AR, Vierstra RD (2002). The *APG8/12*-activating enzyme *APG7* is required for proper nutrient recycling and senescence in *Arabidopsis thaliana*. J Biol Chem.

[40] Makino A, Osmond B (1991). Effects of nitrogen nutrition on nitrogen partitioning between chloroplasts and mitochondria in pea and wheat. Plant Physiol.

[41] Durrett TP, Benning C, Ohlrogge J (2008). Plant triacylglycerols as feedstocks for the production of biofuels. Plant J.

[42] Tevini M, Steinmüller D (1985). Composition and function of plastoglobuli: II. Lipid composition of leaves and plastoglobuli during beech leaf senescence. Planta..

[43] Zhang MP, Zhang CJ, Yu GH (2010). Changes in chloroplast ultrastructure, fatty acid components of thylakoid membrane and chlorophyll a fluorescence transient in flag leaves of a super-high-yield hybrid rice and its parents during the reproductive stage. J Plant Physiol.

[44] Ren G, An K, Liao Y (2007). Identification of a novel chloroplast protein *AtNYE1* regulating chlorophyll degradation during leaf senescence in *Arabidopsis*. Plant Physiol.

[45] Taylor L, Nunes-Nesi A, Parsley K (2010). Cytosolic pyruvate, orthophosphate dikinase functions in nitrogen remobilization during leaf senescence and limits individual seed growth and nitrogen content. Plant J.

[46] Wu S, Li Z, Yang L (2016). *NON-YELLOWING2* (*NYE2*), a close Paralog of *NYE1*, plays a positive role in chlorophyll degradation in Arabidopsis. Mol Plant.

[47] Zheng Y, Qiu Y, Grace CRR, Liu X, Klionsky DJ, Schulman BA (2019). A switch element in the autophagy E2 *Atg3* mediates allosteric regulation across the lipidation cascade. Nat Commun.

[48] Liu L, McNeilage RT, Shi LX, Theg SM (2014). ATP requirement for chloroplast protein import is set by the km for ATP hydrolysis of stromal *Hsp70* in *Physcomitrella patens*. Plant Cell.

[49] Shi LX, Theg SM (2010). A stromal heat shock protein 70 system functions in protein import into chloroplasts in the moss *Physcomitrella patens*. Plant Cell.

[50] Larkin MA, Blackshields G, Brown NP (2007). Clustal W and Clustal X version 2.0. Bioinformatics..

[51] Robert X, Gouet P (2014). Deciphering key features in protein structures with the new ENDscript server. Nucleic Acids Res.

[52] Letunic I, Bork P (2018). 20 years of the SMART protein domain annotation resource. Nucleic Acids Res.

[53] Bailey TL, Boden M, Buske FA (2009). MEME SUITE: tools for motif discovery and searching. Nucleic Acids Res.

[54] Tamura K, Stecher G, Peterson D, Filipski A, Kumar S (2013). MEGA6: molecular evolutionary genetics analysis version 6.0. Mol Biol Evol.

[55] Pu X, Yang L, Liu L (2020). Genome-wide analysis of the MYB transcription factor superfamily in *Physcomitrella patens*. Int J Mol Sci.

[56] Arya D, Kapoor S, Kapoor M (2016). *Physcomitrella patens* DNA methyltransferase 2 is required for recovery from salt and osmotic stress. FEBS J.

[57] Le Bail A, Scholz S, Kost B (2013). Evaluation of reference genes for RT qPCR analyses of structure-specific and hormone regulated gene expression in *Physcomitrella patens* gametophytes. PLoS One.

[58] Suzuki R, Ishimaru T (1990). An improved method for the determination of phytoplankton chlorophyll using N, N-dimethylformamide. Journal of the Oceanographical Society of Japan.

[59] Langmead B, Salzberg SL (2012). Fast gapped-read alignment with bowtie 2. Nat Methods.

[60] Li B, Dewey CN (2011). RSEM: accurate transcript quantification from RNA-Seq data with or without a reference genome. BMC Bioinformatics.

[61] Wang L, Feng Z, Wang X, Wang X, Zhang X (2010). DEGseq: an R package for identifying differentially expressed genes from RNA-seq data. Bioinformatics..

[62] Xu W, Chen Z, Ahmed N, Han B, Cui Q, Liu A (2016). Genome-wide identification, evolutionary analysis, and stress responses of the GRAS gene family in castor beans. Int J Mol Sci.

