# Supplementary material for: Comprehensive analysis of the Ppatg3 mutant reveals that autophagy plays important roles in gametophore senescence in Physcomitrella patens
Source: BMC Plant Biol. 2020 Sep 23;20:440. doi: 10.1186/s12870-020-02651-6 (PMC7513309; doi:10.1186/s12870-020-02651-6)
Supplement: Supplementary file 1 — Additional file 1. [file 12870_2020_2651_MOESM1_ESM.doc]

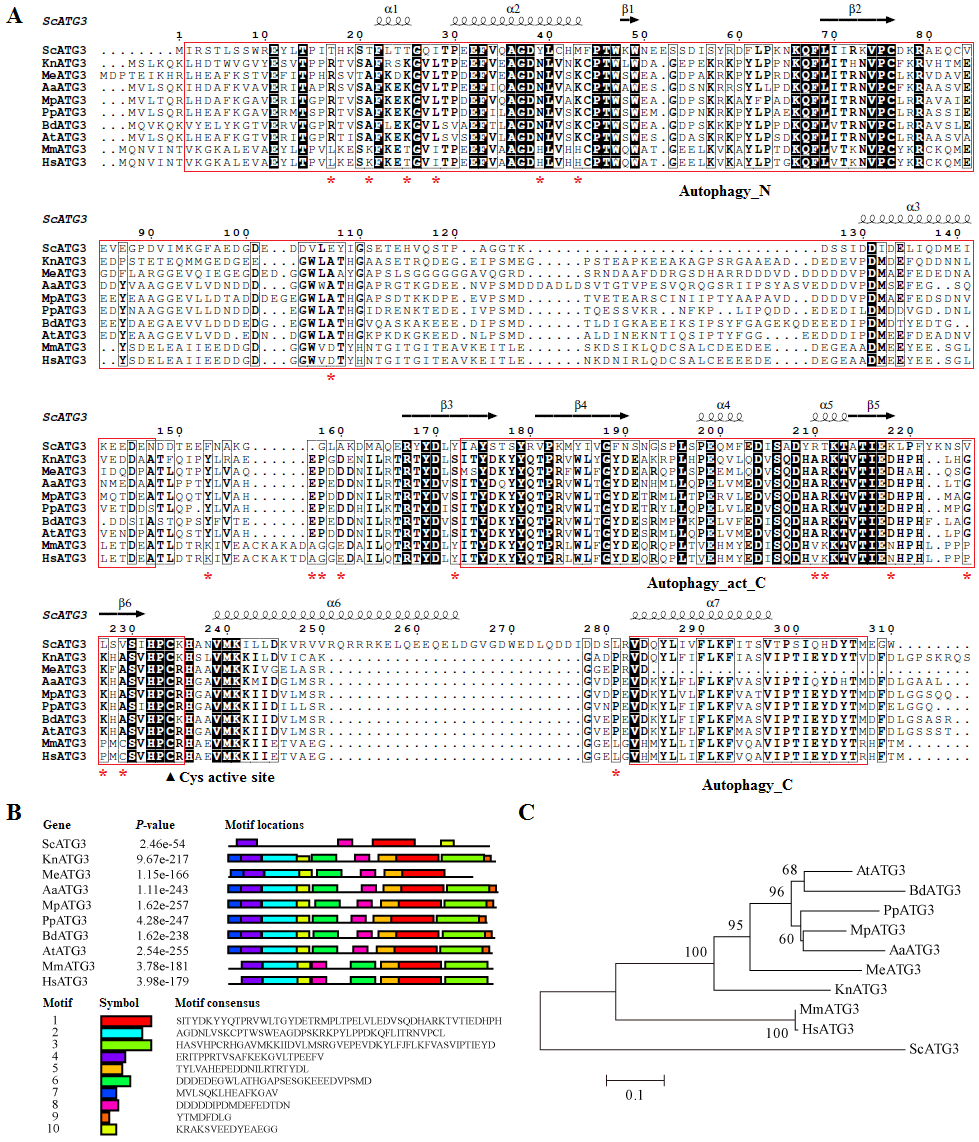


**Additional file 1: Figure S1.** Protein sequence characterization and phylogenetic analysis of PpATG3. (A) Alignment of *PpATG3* protein sequence with homologs from *Saccharomyces cerevisiae*, *Klebsormidium nitens*, *Mesotaenium endlicherianum*, *Anthoceros angustus*, *Marchantia polymorpha*, *Brachypodium distachyon*, *Arabidopsis thaliana*, *Mus musculus* and *Homo sapiens*. Amino acids with more than 70% homology were marked in white frames. The identical amino acids within these ATG3 proteins were highlighted in black shadow. The secondary structure of ScATG3 (PDB ID: 2DYT) was used for analysis. Alpha helices and beta sheets of ATG3s were predicted and shown on the top. Three domains of Autophagy_N, Autophagy_act_C and Autophagy_C were shown in three red frames, respectively. The Cys-234 active site [21] of ATG3 was pointed by a black triangle. Amino acids that are highly conserved among plant species but different from yeast and human/mouse were marked with red asterisks. (B) The conserved motifs in ATG3 proteins were shaded in different colors. The Motif 1, Motif 4, Motif 8 and Motif 10 presented in all the proteins. The Motif 2, Motif 3, Motif 5, and Motif 6 absented in the proteins of yeast. The Motif 7 and Motif 9 presented in the proteins of plants only. (C) Unrooted phylogenetic tree generated by MEGA 6 using a NJ method showing the evolutionary relationship among ATG3 proteins from different organisms.
