# Supplementary material for: Comprehensive analysis of the Ppatg3 mutant reveals that autophagy plays important roles in gametophore senescence in Physcomitrella patens
Source: BMC Plant Biol. 2020 Sep 23;20:440. doi: 10.1186/s12870-020-02651-6 (PMC7513309; doi:10.1186/s12870-020-02651-6)
Supplement: Supplementary file 2 — Additional file 2. [file 12870_2020_2651_MOESM2_ESM.doc]

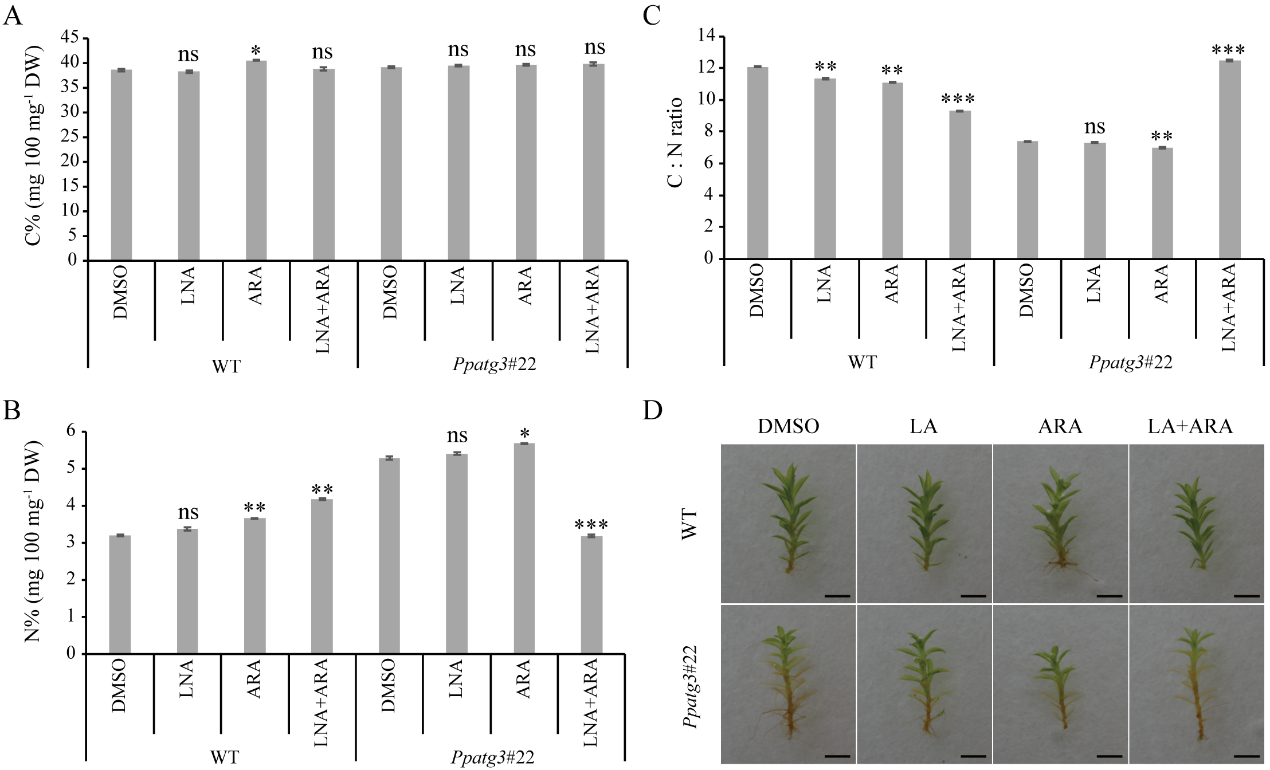


**Additional file 2: Figure S2.** Fatty acid supplementation on C/N ratio of *Ppatg3* knockout plants. (A-C) Comparison of C/N ratio of WT and *Ppatg3* knockout plants after fatty acid supplementation. The 21-day-old plants under normal growth were exogenously treated 7 days for analysis. LA, linoleic acid; ARA, arachidonic acid. DMSO was used as control. Three biological replicates were analyzed and error bars show the mean value ± SD. The asterisks indicate a significant change between the *Ppatg3* and WT plants at (*) p < 0.05, (**) p < 0.01, and (***) p < 0.001. Non-significant differences between the *Ppatg3* and WT plants are denoted (ns). (D) Gametophores senescence phenotype of *Ppatg3* was not decelerated after supplying exogenous fatty acids. The scale bar = 3 mm.
