# Supplementary material for: Comprehensive analysis of the Ppatg3 mutant reveals that autophagy plays important roles in gametophore senescence in Physcomitrella patens
Source: BMC Plant Biol. 2020 Sep 23;20:440. doi: 10.1186/s12870-020-02651-6 (PMC7513309; doi:10.1186/s12870-020-02651-6)
Supplement: Supplementary file 3 — Additional file 3. [file 12870_2020_2651_MOESM3_ESM.doc]

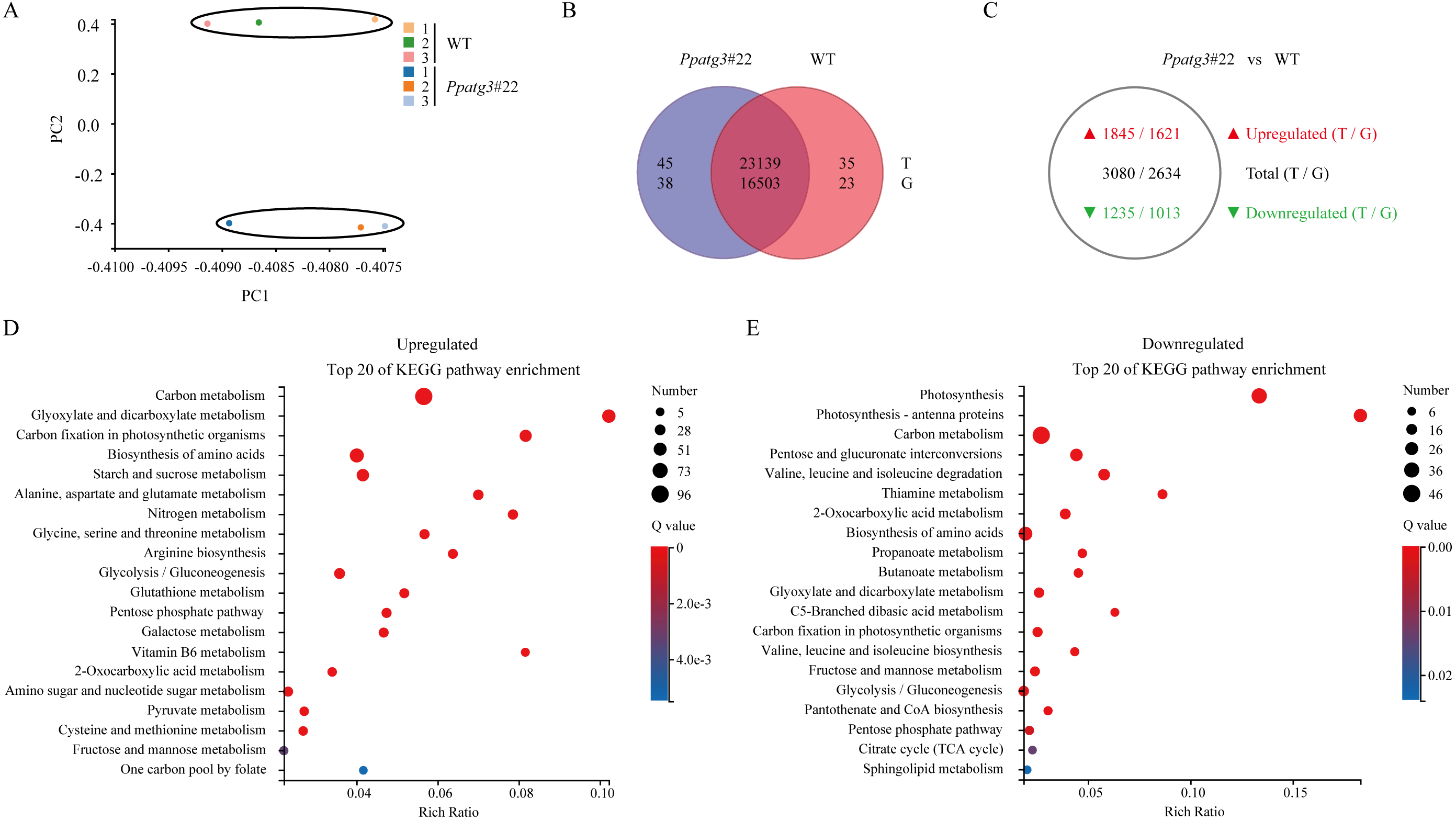


**Additional file 3: Figure S3.** Transcriptome analysis of WT and *Ppatg3*. (A) PCA of 6 samples between WT and *Ppatg3*. (B) The number of co-expressed transcripts/genes and uniquely expressed transcripts/genes between *Ppatg3* mutant and WT. T, transcript; G, gene. (C) The number of DETs/DEGs of *Ppatg3* compared with WT. Up- and downregulated DETs/DEGs shown in red and green lettering, respectively. T, transcript; G, gene. (D and E) Scatter plot for top 20 of KEGG enrichment of up- and downregulated DETs in *Ppatg3* compared with WT, respectively.
