# Supplementary material for: Comprehensive analysis of the Ppatg3 mutant reveals that autophagy plays important roles in gametophore senescence in Physcomitrella patens
Source: BMC Plant Biol. 2020 Sep 23;20:440. doi: 10.1186/s12870-020-02651-6 (PMC7513309; doi:10.1186/s12870-020-02651-6)
Supplement: Supplementary file 9 — Additional file 9 Placeholder Text [file 12870_2020_2651_MOESM9_ESM.doc]

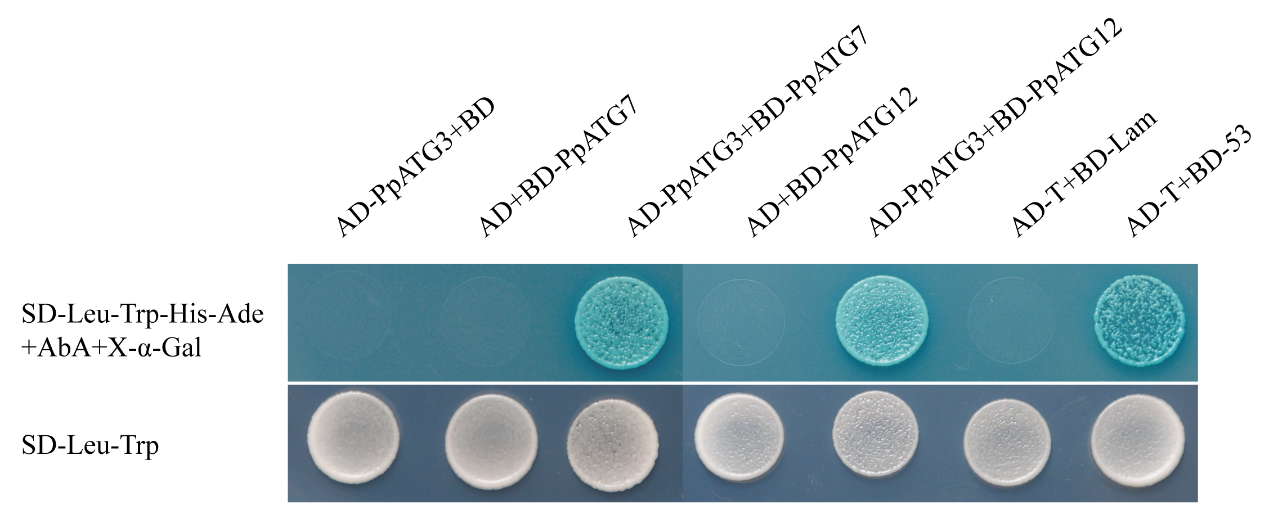


**Additional file 9: Figure S4.** Protein–protein interactions of PpATG3 with PpATG7 and PpATG12 by Y2H. PpATG3 was fused with AD and PpATG7 or PpATG12 was fused with BD. The pGADT7-T (AD-T) was co-transformed with the pGBKT7-Lam (BD-Lam) or pGBKT7-53 (BD-53) to serve as a negative or positive control, respectively. The quadruple dropout medium was supplemented with 40 μg/ml X-α-Gal and 200 ng/ml Aureobasidin A (AbA). The SD-Leu-Trp and SD-Leu-Trp-His-Ade+AbA+X-α-gal medium was used for yeast culture.
