# Supplementary material for: Comprehensive analysis of the Ppatg3 mutant reveals that autophagy plays important roles in gametophore senescence in Physcomitrella patens
Source: BMC Plant Biol. 2020 Sep 23;20:440. doi: 10.1186/s12870-020-02651-6 (PMC7513309; doi:10.1186/s12870-020-02651-6)
Supplement: Supplementary file 10 — Additional file 10. [file 12870_2020_2651_MOESM10_ESM.doc]

**Additional file 10: Table S6.** Primers used in this study.

| **Primer name** | **Sequence (5’-3’)** |
| --- | --- |
| For transient expression experiments | |
| PpATG3-F | GGGGTACCATGGTTTTGTCACAGCGCCTC |
| PpATG3-R | GCTCTAGACTGACCCCCAAGTTCAAAGTC |
| For flank regions amplification | |
| P1 | GGGGTACCAGGCGCTGTAGAAAGGATGAC |
| P2 | CCCAAGCTTAAACCTGAGGCGTGAGAAGAC |
| P3 | GCTCTAGACACTTTTGCGGTCGGTTAGA |
| P4 | CGGGATCCCCACGGGAAAGGAGTATGTCA |
| For gene knockout genotyping | |
| P5 | AAGCGTTGTGCTCCGTTGA |
| P6 | CCACAAGTTAGACACGAATCCC |
| P7 | GACAGAGGATGAAATTGTGCCATC |
| P8 | ACAAGGTACGGTTGTAATGTGCT |
| C1 | TGCCTCGTCTTGGAGTTCATTC |
| C2 | TATCAGGACATAGCGTTGGC |
| C3 | GCCGAATATCATGGTGGAAAATGG |
| C4 | ACCGTAAAGCACGAGGAAGC |
| PpUbiquitin-F | ACTACCCTGAAGTTGTATAGTTCGG |
| PpUbiquitin-R | CAAGTCACATTACTTCGCTGTCTAG |
| PpAdePRT-F | AGTATAGTCTAGAGTATGGTACCG |
| PpAdePRT-R | TAGCAATTTGATGGCAGCTC |
| For Y2H analysis | |
| AD-PpATG3-F | GAATTCATGGTTTTGTCACAGCGCCTC |
| AD-PpATG3-R | GGATCCTCACTGACCCCCAAGTTCAAAG |
| BD-PpATG7-F | CATATGATGGGGATTACTTCGCCGC |
| BD-PpATG7-R | GTCGACTCAAAAGTCTGCATCGTCGTCC |
| BD-PpATG12-F | CATATGATGTCAACTTCGGCGGAATCGC |
| BD-PpATG12-R | GTCGACTTAACCCCAAGCCATAGAGCAGG |
| For gene expression analysis | |
| PpAdePRT-qF | ATTGCATTGAGATGCACGTT |
| PpAdePRT-qR | CCCTTCAGGTCTACCAGCTC |
| Pp3c17_23030-qF | AAGATCACCTTGGCCATTTC |
| Pp3c17_23030-qR | AGATTCGCGATCAAGTTGTG |
| Pp3c1_5000-qF | GGGGTTGGATGCAGATAGTT |
| Pp3c1_5000-qR | GGAGTCAGAAGGCTGAAAGG |
| Pp3c5_22540-qF | CCTCGTAGCTTGTCCCTAGC |
| Pp3c5_22540-qR | CCTTCCCGAATGTGAAAACT |
| Pp3c21_10860-qF | GCAGTGACACCGATTTCAAG |
| Pp3c21_10860-qR | TTTTCTTGTCCTGCGTGAAG |
| Pp3c7_3360-qF | CGCCATGAACGAAGCTATTA |
| Pp3c7_3360-qR | ACCGTAGCTACCACCACCTC |
| Pp3c7_6560-qF | CGGTGGAGAAGAATTCAGGT |
| Pp3c7_6560-qR | CTCATCGGCGAAAGTAACAA |
| Pp3c19_20120-qF | GCGGTCGCTACTGGAATAAT |
| Pp3c19_20120-qR | AGGCAAATGTGATGACCAGA |
| Pp3c22_9450-qF | GAGGGGTATTGAGCGAAGAG |
| Pp3c22_9450-qR | AGCCCACTTCGATACAGCTT |
| Pp3c4_7680-qF | TCCCGTCTGCTATGTCTCTG |
| Pp3c4_7680-qR | GGCGTTTAGGCTTGAGATTC |
| Pp3c21_5770-qF | GCAGTCGAATCGAGAGTCTG |
| Pp3c21_5770-qR | GTGCATCAAGCTGCTGTTTT |
| Pp3c9_10620-qF | AAGGTTGTAATGGCCAGGAG |
| Pp3c9_10620-qR | GACCCCTACGAGCAAACAAT |
| Pp3c15_13040-qF | CAGGAGTTCGATACCGTTGA |
| Pp3c15_13040-qR | GGAGTGGATGCTCCATTTTT |
| Pp3c7_5390-qF | GTGTTCAATCAGTGGCAACC |
| Pp3c7_5390-qR | ATCCACAGAAAGAGGGATGG |
| Pp3c11_19850-qF | GTATTTTGGCGGAGAGGAAG |
| Pp3c11_19850-qR | GACAAAATGCGATTCCACAC |
| Pp3c24_15420-qF | GATCGTGCTGTGAAGCAAAT |
| Pp3c24_15420-qR | GCTGGACGGGATTTCTGTAT |
| Pp3c21_8940-qF | AGGACAGCGAGGTGATTCTT |
| Pp3c21_8940-qR | CTCCTCATCCACGACCTCTT |
| Pp3c21_8810-qF | AGGACAGCGAGGTGATTCTT |
| Pp3c21_8810-qR | CTCCTCATCCACGACCTCTT |
| Pp3c18_10780-qF | GTTGACTGGAAAGCACGAGA |
| Pp3c18_10780-qR | GTCGTGTATGCGATCATTCC |
| Pp3c18_10760-qF | GTTGACTGGAAAGCACGAGA |
| Pp3c18_10760-qR | GTCGTGTATGCGATCATTCC |
| Pp3c8_17940-qF | TAACGTAGACCGAGCTGTCG |
| Pp3c8_17940-qR | ATCATTTGCTTCACCAACCA |
| Pp3c14_9410-qF | CATTTCGGAGACGGAAGAAT |
| Pp3c14_9410-qR | AAGGGGTGACGACCTGTAAG |
