# Supplementary figures and images for: Comprehensive analysis of the Ppatg3 mutant reveals that autophagy plays important roles in gametophore senescence in Physcomitrella patens
Source: BMC Plant Biol. 2020 Sep 23;20:440. doi: 10.1186/s12870-020-02651-6 (PMC7513309; doi:10.1186/s12870-020-02651-6)

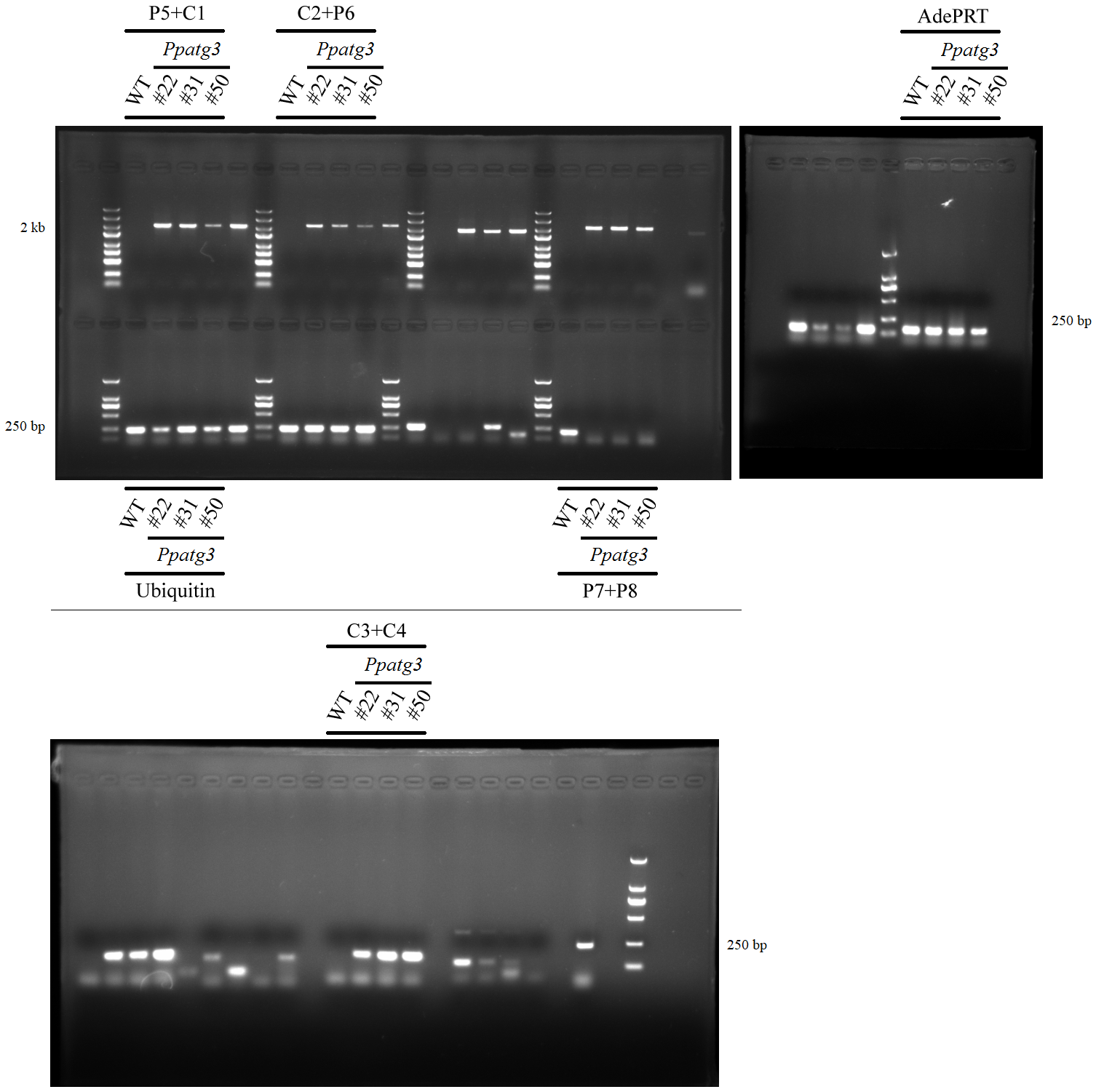


**Additional file 11: Figure S5.** Original and uncropped gels for Figure 1C.

Supplement: Supplementary file 11 — Additional file 11. [file 12870_2020_2651_MOESM11_ESM.doc]
